# Human iPSC-derived pericyte-like cells carrying APP Swedish mutation overproduce beta-amyloid and induce cerebral amyloid angiopathy-like changes

**DOI:** 10.1186/s12987-024-00576-y

**Published:** 2024-09-27

**Authors:** Ying-Chieh Wu, Šárka Lehtonen, Kalevi Trontti, Riitta Kauppinen, Pinja Kettunen, Ville Leinonen, Markku Laakso, Johanna Kuusisto, Mikko Hiltunen, Iiris Hovatta, Kristine Freude, Hiramani Dhungana, Jari Koistinaho, Taisia Rolova

**Affiliations:** 1 Neuroscience Center, University of Helsinki, 00014 Helsinki, Finland; 2 A.I.Virtanen Institute for Molecular Sciences, University of Eastern Finland, 70211 Kuopio, Finland; 3 NeuroCenter, Kuopio University Hospital, Kuopio, Finland; 4 Institute of Clinical Medicine, University of Eastern Finland, Kuopio, Finland; 5 Department of Medicine and Clinical Research, Kuopio University Hospital, Kuopio, Finland; 6 Institute of Biomedicine, University of Eastern Finland, Kuopio, Finland; 7 SleepWell Research Program, Faculty of Medicine, University of Helsinki, Helsinki, Finland; 8 Department of Veterinary and Animal Sciences, University of Copenhagen, 1870 Frederiksberg, Denmark; 9 Helsinki Institute of Life Science, University of Helsinki, 00014 Helsinki, Finland; 10 Drug Research Program, Division of Pharmacology and Pharmacotherapy, University of Helsinki, 00014 Helsinki, Finland

**Keywords:** Pericytes, Vascular dysfunction, Alzheimer’s disease, Cerebral amyloid angiopathy, iPSCs

## Abstract

**Background:**

Patients with Alzheimer's disease (AD) frequently present with cerebral amyloid angiopathy (CAA), characterized by the accumulation of beta-amyloid (Aβ) within the cerebral blood vessels, leading to cerebrovascular dysfunction. Pericytes, which wrap around vascular capillaries, are crucial for regulating cerebral blood flow, angiogenesis, and vessel stability. Despite the known impact of vascular dysfunction on the progression of neurodegenerative diseases, the specific role of pericytes in AD pathology remains to be elucidated.

**Methods:**

To explore this, we generated pericyte-like cells from human induced pluripotent stem cells (iPSCs) harboring the Swedish mutation in the amyloid precursor protein (APPswe) along with cells from healthy controls. We initially verified the expression of classic pericyte markers in these cells. Subsequent functional assessments, including permeability, tube formation, and contraction assays, were conducted to evaluate the functionality of both the APPswe and control cells. Additionally, bulk RNA sequencing was utilized to compare the transcriptional profiles between the two groups.

**Results:**

Our study reveals that iPSC-derived pericyte-like cells (iPLCs) can produce Aβ peptides. Notably, cells with the APPswe mutation secreted Aβ1-42 at levels ten-fold higher than those of control cells. The APPswe iPLCs also demonstrated a reduced ability to support angiogenesis and maintain barrier integrity, exhibited a prolonged contractile response, and produced elevated levels of pro-inflammatory cytokines following inflammatory stimulation. These functional changes in APPswe iPLCs correspond with transcriptional upregulation in genes related to actin cytoskeleton and extracellular matrix organization.

**Conclusions:**

Our findings indicate that the APPswe mutation in iPLCs mimics several aspects of CAA pathology in vitro, suggesting that our iPSC-based vascular cell model could serve as an effective platform for drug discovery aimed to ameliorate vascular dysfunction in AD.

**Supplementary Information:**

The online version contains supplementary material available at 10.1186/s12987-024-00576-y.

## Background

Alzheimer’s disease (AD) is a progressive neurodegenerative disorder that accounts for 60–70% of dementia cases. Familial AD is caused by gene mutations in the amyloid precursor protein (*APP*) or presenilin-1 and -2 (*PSEN1/2*) genes [1] that enhance brain deposition of beta-amyloid (Aβ) peptides resulting in amyloid pathology. While most cases of AD are sporadic, investigating familial AD can offer valuable insights, as both familial and sporadic AD share similar pathological features.

Although AD research has focused chiefly on neurons, neuronal function largely depends on cerebral blood flow (CBF), providing an adequate supply of oxygen and glucose. Recent imaging studies suggest that hypoperfusion is one of the earliest disease-associated changes in the AD brain, promoting cognitive impairment independently from parenchymal amyloid deposition [2–4]. Cerebral amyloid angiopathy (CAA), the accumulation of Aβ in brain vasculature, is considered a fundamental underlying cause of vascular dysfunction in AD, and it is also a risk factor for hemorrhagic stroke [5]. Pericytes and smooth muscle cells (SMCs) are mural cells that wrap around brain capillaries and form arterial vessel walls, respectively. They play an essential role in vascular stability and CBF regulation [6]. CAA promotes the degeneration of endothelial cells, pericytes and SMCs and compromises the integrity of the blood–brain barrier (BBB) [5, 7, 8]. A single-nucleus RNA-sequencing analysis of AD patient brains showed that AD risk genes are highly expressed in vasculature-associated cell types [9]. Similar findings emerged in the aged mouse brain, where vascular cells exhibited senescent phenotypes or displayed increased expression levels of AD risk genes [10].

To address the impact of genetic mutations associated with familial AD on pericyte function, we generated pericyte-like cells (iPLCs) from three human iPSC lines carrying the KM670/671NL mutation in APP (APPswe), along with seven control lines from healthy individuals (Table 1). Our iPLCs express characteristic pericyte markers, promote a formation of complex tube structure in iPSC-derived endothelial cells (iECs) and decrease iEC layer permeability, thus demonstrating several normal pericyte functions. However, the presence of APPswe mutation impaired the pericyte functions. Therefore, human iPSC-derived vascular cells have the potential to serve as a model for investigating vascular dysfunction in neurodegenerative diseases.

**Table 1:** iPSC lines used in this study

Represent symbol	Cell line	Sex	Age at Biopsy	APP genotype	APOE genotype	Status when sample taken	References
	Ctrl1	F	77	Control	ɛ3/ɛ3	Healthy	Figure S1
	Ctrl2	F	77	Control	ɛ3/ɛ3	Healthy	Figure S1
	Ctrl3	F	30	Control	ɛ3/ɛ3	Healthy	[11]
	Ctrl4	M	63	Control	ɛ3/ɛ3	Healthy	[12]
	Ctrl5	M	66	Control	ɛ3/ɛ3	Healthy	[13]
	Ctrl6	M	64	Control	ɛ3/ɛ3	Healthy	[14]
	Ctrl7	M	64	Control	ɛ3/ɛ4	NPH w/o amyloid pathology	Figure S1
	Ctrl iAstrocyte	F	44	Control	ɛ3/ɛ3	Healthy	[15]
	AD1	F	58	APPswe	ɛ3/ɛ3	AD	[16]
	AD2	F	30	APPswe	ɛ3/ɛ3	Pre-symptomatic AD	[17]
	AD3	M	15–19	APPswe	ɛ3/ɛ4	Healthy control introduced APPswe	[18]

## Materials and methods

### Patients and iPSCs

AD iPSC lines were generated from two individuals harboring the APPswe mutation: AD1 was diagnosed with AD, while AD2 was pre-symptomatic without a clinical diagnosis. The third APPswe line, AD3, was generated by introducing KM670/671NL mutation in a healthy male individual using CRISPR-Cas9 [18] and was kindly provided to us by Dr. Kristine Freude. We used altogether six controls from healthy male and female individuals in this study. To reduce the complexity of the genetic background and investigate the phenotype developing from the APPswe mutation, the selected controls (Ctrl1-6) and APPswe mutation (AD1-2) individuals were all carrying apolipoprotein (APOE) ε3/ε3, the AD risk-neutral allele. However, AD3 was carrying ε3/ε4 as its parental line. Therefore, we introduced Ctrl7, which carried the APOE ε3/ε4 allele and came from a patient diagnosed with normal pressure hydrocephalus (NPH) without amyloid pathology, as an additional control for AD3 (Table 1). All iPSC lines expressed pluripotency markers, exhibited a normal karyotype, and could form embryoid bodies and differentiate into three germ layers. The iPSC lines included in individual experiments are listed in Table S1.

### Generation and maintenance of iPSCs

Dermal biopsies were collected with informed consent and approval from the Committee on Research Ethics of Northern Savo Hospital District (license no. 123/2016). Fibroblasts were expanded as described previously [19]. Then, somatic cells were reprogrammed to iPSCs with CytoTune-iPS 2.0 Sendai Reprogramming Kit (Thermo Fisher Scientific) as described [11]. Ctrl1 and Ctrl2 lines were reprogrammed at the Biomedicum Stem Cell Centre core facility, University of Helsinki, using CRISPR activators [20, 21]. Briefly, fibroblasts were detached as single cells from the culture plates with TrypLE Select (Gibco, Thermo Fisher Scientific) and electroporated using the Neon transfection system (Invitrogen, Thermo Fisher Scientific) using CRISPRa plasmids [20]. Electroporated fibroblasts were plated on Matrigel-coated plates (growth factor reduced; Corning; 1:200) immediately after the transfections. The medium was changed every other day, and on day 4, the fibroblast medium was changed to a 50:50 ratio of fibroblast medium and stem cell medium (DMEM/F12 with 1% GlutaMAX supplemented with 20% KnockOut Serum Replacement, 0.0915 mM 2-mercaptoethanol, 1% Non-Essential Amino Acids (NEAA; all from Gibco, Thermo Fisher Scientific), 6 ng/mL basic fibroblast growth factor (bFGF; Merck), and 0.25 mM NaB). iPSCs were maintained on Matrigel-coated plates in Essential 8 Medium (E8; Gibco, Thermo Fisher Scientific). 0.5 mM EDTA (Thermo Fisher Scientific) was used to detach the cells while passaging, and 5 µM Y27632 ROCK inhibitor (Selleckchem) was applied into the culture to enhance attachment when thawing.

### Differentiation of iPLCs

The iPLCs differentiation protocol was adapted from Blanchard et al., 2020 [22] with slight modifications. On day 0, iPSCs were dissociated to single cells by StemPro Accutase (Thermo Fisher Scientific) and replated on Matrigel-coated plates at a density of 2 × 10^4^ cells/cm^2^ in E8 medium with 10 µM Y27632. On day 1, the medium was switched to N2B27 medium (1:1 DMEM/F12 and Neurobasal medium supplemented with 1% GlutaMAX, 2% B-27, 1% N2 (all from Gibco, Thermo Fisher Scientific), 100 µg/ml Primocin (InvivoGen), and 50 µM 2-mercaptoethanol (Merck)) supplemented with 25 ng/mL bone morphogenetic protein (BMP)-4 (PeproTech, Thermo Fisher Scientific) and 8 µM CHIR99021 (Cayman Chemical) for 4 days for mesoderm specification. The medium was changed every other day. On day 5, the medium was switched to N2B27 medium supplemented with 10 ng/mL platelet-derived growth factor (PDGF)-BB and 2 ng/mL transforming growth factor (TGFβ)-3 (both from PeproTech, Thermo Fisher Scientific) for two days to induce differentiation toward iPLCs. The iPLCs were maintained in N2B27 medium without supplements until Day 21 after the initiation of differentiation. On Day 21–23, the iPLCs were either seeded or collected for experiments. Some pilot experiments were conducted at slightly later time points, but none extended beyond Day 35.

### Differentiation of iECs

The iEC differentiation protocol was adapted from K. Wang et al. [23]. Initially, the iPSC lines were transduced with E26 transformation-specific variant (ETV) 2 under the control of a doxycycline-inducible promoter (Tet-On system). iPSCs were seeded in E8 medium with 10 µM Y27632 at 7 × 10^3^ cells/cm^2^ and incubated overnight. The next day, cells were transduced with 2 × 10⁶ TU/mL of lentivirus in E8 medium for 24 h. Then the virus-containing medium was replaced, and cells were allowed to recover for 72 h before selection with 400 µg/mL G418 (InVivoGen) for 7 days. The pInducer20-ETV2 plasmid was developed by the Genome Biology Unit, University of Helsinki. Biomedicum Virus Core, University of Helsinki, produced lentivirus particles.

For iECs differentiation, on day 0, iPSCs transduced with ETV2 were dissociated into single cells using StemPro Accutase and plated onto Matrigel-coated plates at a density of 2.2 × 10^4^ cells/cm^2^ in E8 medium supplemented with 10 µM Y27632. On day 1, the culture medium was changed to S1 medium (DMEM/F12 supplemented with 1% GlutaMAX, 60 μg/mL L-Ascorbic acid, 0.5% penicillin/streptomycin, and 6 µM CHIR99021. On day 3, the S1 medium was replaced with StemPro medium (StemPro-34 SFM) supplemented with 50 ng/ml vascular endothelial growth factor (VEGF)-A, 50 ng/mL bFGF, 10 ng/mL epidermal growth factor (EGF; all growth factors from PeproTech, Thermo Fisher Scientific), 10 μM SB431542, and 2 μM doxycycline hyclate (BioGems). On day 5, the medium was further changed to Human Endothelial SFM (Thermo Fisher Scientific) supplemented with 5% KnockOut serum replacement (Thermo Fisher Scientific), 10 ng/mL bFGF, 5 ng/mL EGF, and 0.5 ng/mL VEGF-A. Subsequently, the cells were passaged in this medium and used for experiments within one week.

### Differentiation of iAstrocytes

The iAstrocytes were differentiated as described [15]. In short, neural induction was achieved by culturing the iPSCs in neural differentiation medium (NDM, 1:1 DMEM/F12 and Neurobasal medium, 1% B-27 without vitamin A, 0.5% N2, 1% GlutaMAX, and 0.5% penicillin/streptomycin (50 IU/50 μg/mL)) supplemented with dual SMAD inhibitors 10 μM SB431542 and 200 nM LDN193189 (both from Merck) for 12 days until rosette-like structures appeared. Then, the medium was switched to NDM supplemented with 20 ng/mL bFGF for 2 to 3 days to expand the rosettes. The areas with rosettes were lifted and cultured in ultra-low attachment plates (Corning) with NDM for two days for sphere formation. Next, spheres were cultured in astrocyte differentiation medium (ADM, DMEM/F12, 1% N2, 1% GlutaMAX, 1% NEAA, 0.5% penicillin/streptomycin, and 0.5 IU/mL heparin (Leo Pharma)) supplemented with 10 ng/mL bFGF and 10 ng/mL EGF (PeproTech; ThermoFisher Scientific). Spheres were cultured in ADM medium for 6 to 9 months to get pure astroglial cultures. To maturate astrocyte progenitors before the assay, spheres were dissociated with StemPro Accutase and replated on Matrigel-coated plates in ADM supplemented with 10 ng/mL ciliary neurotrophic factor (CNTF; PeproTech, Thermo Fisher Scientific) and 10 ng/mL BMP-4 for 7 to 10 days.

### Differentiation of iNeurons

The iNeurons were obtained using the combination of neurogenin (NGN)2 overexpression with dual SMAD and WNT inhibition as described previously [13]. In short, on day 0, a 60–70% confluent plate of iPSCs transduced with NGN2 under Tet-On promoter was exposed to 2 μg/mL doxycycline in E8 medium. On day 1, the medium was switched to N2 medium (DMEM/F12, 1 × N2, 1 × GlutaMAX, 0.3% glucose) supplemented with 2 μg/mL doxycycline, dual SMAD inhibitors (100 nM LDN193189, 10 μM SB431542B) and 2 μM WNT pathway inhibitor XAV939 (BioGems). On day 2, the concentration of doxycycline and dual SMAD inhibitors was halved, and 5 μg/mL puromycin (MP biomedicals) was added for the selection of NGN*2*-transduced cells. After removing puromycin and dead cells on day 3, the differentiation continued in the N2 medium with a full concentration of supplements as on day 1. On day 4, emerging neurons were plated on poly-D-lysine (Thermo Fisher Scientific) and laminin (from a mouse Engelbreth-Holm-Swarm sarcoma; ~ 1.5 μg/cm2; Merck)-coated surfaces. The medium was then changed to Neurobasal supplemented with 1% GlutamMAX, 2% B-27, 50 μM NEAA, 0.3% glucose, and neurotrophic factors (10 ng/mL of glial cell-derived neurotrophic factor (GDNF), brain-derived neurotrophic factor (BDNF) and CNTF (all from PeproTech, Thermo Fisher Scientific). Proliferation was halted on day 7 with an overnight treatment of 10 μM floxuridine (Bio-Techne), and cells matured for three weeks, with medium changes three times weekly.

### Immunocytochemistry

Cells were washed with PBS and fixed with 3.7% formaldehyde (Merck) for 20 min at room temperature (RT). Afterward, cells were permeabilized with 0.3% Triton X-100 (Merck) and blocked with 5% normal goat serum (Merck) in PBS for 1 h at RT. Primary antibodies, diluted in PBS with 5% NGS, were applied and incubated overnight at 4 °C. Following three washes with PBS, secondary antibodies were applied and incubated for 1 h at RT. Nuclei were stained with 1 µg/mL DAPI (Sigma) for 10 min at RT. Primary and secondary antibodies are listed in Table 2. Coverslips were mounted on glass slides using Fluoromount-G™ mounting medium (Thermo Fisher Scientific). Images were visualized by an EVOS microscope (Thermo Fisher Scientific) with 4 × and 10 × objectives. Brightness and contrast were adjusted using ImageJ software (National Institutes of Health).

**Table 2:** Primary and secondary antibodies used for ICC

Target	Dilution	Source	Catalog no
Mouse anti- PDGFRβ	1:100	R&D Systems	AF385-SP
Mouse anti-Chondroitin Sulfate Proteoglycan (NG2)	1:100	Merck	MAB2029
Rabbit anti-Actin, α-Smooth Muscle + ACTG2	1:300	Abcam	ab32575
Mouse anti-Oct4	1:400	EMD Millipore	MAB4401
Mouse anti-CD31	1:500	Agilent Dako	M0823
Goat anti-Nanog	1:100	R&D Systems	AF1997
Mouse anti-TRA-1–81	1:200	EMD Millipore	MAB4381
Mouse anti-SSEA4	1:400	EMD Millipore	MAB4304
Mouse anti-α-SMA	1:300	Sigma	A5228
Mouse anti-B -III-tubulin	1:1000	Covance	MMS-435P
Mouse anti-AFP	1:300	Sigma	A8452
Goat anti-Mouse IgG (H + L), Alexa Fluor™ 488	1:300	Thermo Fisher Scientific	A-11001
Goat anti-Rabbit IgG (H + L), Alexa Fluor™ 568	1:300	Thermo Fisher Scientific	A-11011

### qRT-PCR

Total RNA was extracted from iPSCs, iPLCs, iECs, and iAstrocytes using the RNeasy Mini Kit (Qiagen) following the manufacturer’s instructions. RNA concentrations were measured by the SimpliNano Spectrophotometer (Biochrom). RNA was subsequently reverse transcribed to complementary DNA (cDNA) using the Maxima Reverse Transcriptase in the presence of RiboLock RNase Inhibitor, dNTP Mix, and Random Hexamer Primer (all from Thermo Fisher Scientific). mRNA levels were quantified via quantitative RT-PCR using TaqMan assay probes (listed in Table 3) with Maxima Probe/ROX qPCR Master Mix (Thermo Fisher Scientific) on the CFX96 Real-Time PCR System (Bio-Rad). *GAPDH* served as the normalization control. Table 4

**Table 3:** primers assay mixes used for mRNA expression studies

Gene	Identifier	Source
*PDGFRB*	Hs01019589_m1	TaqMan, Thermo Fisher Scientific
*DES*	Hs00157258_m1	TaqMan, Thermo Fisher Scientific
*LAMA2*	Hs00166308_m1	TaqMan, Thermo Fisher Scientific
*DLC1*	Hs00183436_m1	TaqMan, Thermo Fisher Scientific
*PDE7B*	Hs01054008_m1	TaqMan, Thermo Fisher Scientific
*CD248*	Hs00535586_s1	TaqMan, Thermo Fisher Scientific
*ACTA2*	Hs00426835_g1	TaqMan, Thermo Fisher Scientific
*APP*	Hs00169098_m1	TaqMan, Thermo Fisher Scientific
*LRP1*	Hs00233856_m1	TaqMan, Thermo Fisher Scientific
*BACE1*	Hs01121195_m1	TaqMan, Thermo Fisher Scientific
*VEGFA*	Hs00900055_m1	TaqMan, Thermo Fisher Scientific
*ITGA7*	Hs01056475_m1	TaqMan, Thermo Fisher Scientific
*COL1A1*	Hs00164004_m1	TaqMan, Thermo Fisher Scientific
*EDNRA*	Hs03988672_m1	TaqMan, Thermo Fisher Scientific
*EDNRB*	Hs00240747_m1	TaqMan, Thermo Fisher Scientific
*NANOG*	Hs02387400_g1	TaqMan, Thermo Fisher Scientific
*SOX2*	Hs01053049_s1	TaqMan, Thermo Fisher Scientific
*LIN28A*	Hs00702808_s1	TaqMan, Thermo Fisher Scientific
*CDH5*	Hs00901465_m1	TaqMan, Thermo Fisher Scientific
*GAPDH*	Hs99999905-m1	TaqMan, Thermo Fisher Scientific

**Table 4:** Primary and secondary antibodies used for WB

Target	Dilution	Source	Catalo no
Rabbit anti-Phospho-p44/42 MAPK (Erk1/2)	1:1000	Cell Signaling	9101S
Rabbit anti-p44/42 MAPK (Erk1/2)	1:1000	Cell Signaling	9102S
Rabbit anti-Occludin	1:500	Thermo Fisher Scientific	71–1500
Rabbit anit-ZO-1	1:500	Thermo Fisher Scientific	600–401-GU7
Mouse anti-α-Tubulin	1:1000	Cell Signaling	3873S
Mouse anti-α-SMA	1:2000	Sigma	A5228
Mouse anti-APP	1:1000	Sigma	MAB348
Goat anti-Rabbit IgG (H + L), HRP	1:10,000	Thermo Fisher Scientific	A16096
Rabbit anti-mouse IgG –Peroxidase	1:80,000	Merck	A9044

### Western blot

To track the changes in Erk and Akt phosphorylation after ET-1 treatment, 2 × 10^5^ iPLCs were seeded on Matrigel-coated 4-well plates. One day post-seeding, cells were exposed to 30 nM ET-1. At intervals of 15, 30, 45, 60, and 90 min post-exposure of ET-1, cells were lysed using radioimmunoprecipitation assay buffer (RIPA) with Halt™ protease inhibitor and Halt™ phosphatase inhibitor cocktails (all from Thermo Fisher Scientific). Protein concentrations were determined by Pierce™ BCA Protein Assay Kit (Thermo Fisher Scientific). Subsequently, 5 µg of protein per sample were separated under reducing conditions on 10% SDS-PAGE pre-cast gels (Bio-Rad). The proteins were then transferred to polyvinylidene difluoride (PVDF) membranes using the Trans-Blot Turbo™ Transfer Starter System and Midi PVDF kit (both from Bio-Rad). Membranes were blocked for 1 h at RT with 5% non-fat dry milk (NFDM) in Tris-Buffered Saline with 0.05% Tween 20 (TBST). Subsequently, membranes were incubated overnight at 4 °C with primary antibodies diluted in 5% bovine serum albumin (BSA) with 0.02% sodium azide in TBST. After washing in TBST, membranes were incubated with horseradish peroxidase (HRP)-conjugated secondary antibodies for 1 h at RT. Protein bands were visualized using the Pierce™ ECL or Pierce™ ECL Plus Western Blotting Substrate (Thermo Fisher Scientific) and imaged on a G:BOX Chemi imaging system (Syngene). Relative protein levels were quantified with ImageJ and normalized to α-tubulin.

To detect α-SMA and APP protein levels, 1 × 10^6^ cells were replated on Matrigel-coated 6-well plates and lysed the next day in RIPA buffer containing protease inhibitors. For detecting Occludin and ZO-1 proteins, cells were harvested from Transwell inserts using the same lysis protocol. The subsequent steps were as described above, with protein levels normalized to GAPDH.

### 2D tube formation assay

Black-walled, optically clear-bottom 96-well plates (PerkinElmer) were pre-coated with 50 µl of Matrigel and incubated at 37 °C for 30 min to allow Matrigel polymerization. Afterward, 3 × 10^4^ iECs and 1 × 10^4^ iPLCs, or 3 × 10^4^ iPLCs for iPLC-only cultures, were stained with 100 nM Calcein AM (Cayman Chemical) for 15 min at 37 °C and seeded on top of the Matrigel layer in a 1:1 mixture of the endothelial medium and N2B27 medium. Images were captured every 2 h using Incucyte S3 (Sartorius) whole well module with 4 × magnification, phase contrast, and green channel settings. The analysis of total length, number of segments and meshes was performed using images taken 6 h post-replating with ImageJ Angiogenesis Analyzer module. Images were cropped to exclude shadowed regions, and the analyzed area of each image was divided by the total well area to calculate the percentage of the analyzed area relative to the whole well. Results were then multiplied by this percentage to normalize for differences in the regions analyzed.

### Permeability assay

iPLCs were seeded at a density of 1.5 × 10^5^/cm^2^ on basolateral sides of 24-well Transwell inserts (0.4 µm pore size, 0.33 cm^2^ growth area; Corning) coated with 100-fold diluted Matrigel. After 24 h, the apical side of the Transwell inserts was coated with 100-fold diluted Matrigel and incubated for 2 h at 37 °C. iECs were then dissociated into single-cell suspensions using StemPro Accutase for 5 min. Following dissociation, iECs were replated on the apical side of Transwell insert membranes at a density of 4.5 × 10^5^/cm^2^, which worked best in our hands and which is comparable to the densities used by Blanchard and coworkers [22]. Permeability assays were performed 7 days following the replating of iECs. 4 kDa and 70 kDa dextran labeled with Alexa 488, and Texas red fluorophores, respectively (both from Sigma), were diluted in the medium to reach a working concentration of 0.5 mg/mL. Standard curves were established, ranging from 0.5 mg/mL to 160 ng/mL through five-fold serial dilutions. For the assay, 900 µl of fresh medium was added to the bottom chamber, and 300 µl 0.5 mg/mL dextran-medium mixture was introduced to the upper chamber. After a 1-h incubation at 37 °C, 100 µl of medium was sampled from the bottom well, and the mean fluorescent intensity (MFI) was quantified using the FLUOstar Omega spectrometer (BMG Labtech). The concentrations of dextran on the basolateral side were determined by interpolating MFI values against a pre-established standard curve. Then, the permeability coefficient (*P*_*e*_) was calculated using a method previously described in the literature [24, 25]. The transferred volume (mL) of dextran, which diffused from the apical to the basolateral chamber, was derived from the initial concentration of dextran on the apical side ([*C*]_*A*_) and the final concentration on the basolateral side ([*C*]_*B*_) of the chamber: transferred volume (mL) = [*C*]_*B*_ × *V*_*B*_/[*C*]_*A*_, where *V*_*B*_ represents the volume of the basolateral chamber. The permeability surface-area product (*PS*), measured in cm^3^/sec or mL/sec, was calculated by plotting the transferred volume against the reaction time. The *PS* values were then divided by the surface area of the Transwell inserts to compute the permeability coefficient (*P*_*e*_ in cm/sec).

### Aβ secretion and uptake

iPLCs were seeded at a density of 1.5 × 10^5^/cm^2^ on 96-well plates coated with 200-fold diluted Matrigel. Following six days of cultivation without medium change, the medium was collected, and the levels of Aβ1-40 and Aβ1-42 were quantified using ELISA kits according to the manufacturer’s instructions (R&D Systems). Results were normalized to the total protein concentration in the cell lysates, determined by the Pierce BCA protein assay (Thermo Fisher Scientific).

Fluorophore-conjugated Aβ1–42 (HiLyte Fluor 488-labeled, AnaSpec) was prepared by reconstituting 0.1 mg in 50 µl of water. Then, the solution was sonicated and further diluted to a 5 µM concentration in N2B27 medium. Cells were incubated with 5 µM of fluorophore-conjugated Aβ1–42 in N2B27 medium for 24 h. Following incubation, cultures were washed three times with PBS to remove any unbound residues. Nuclei were stained using 1 µg/mL Hoechst (Thermo Fisher Scientific). The percentages of Aβ1–42-positive cells were quantified from images taken with the EVOS microscope using a 10 × objective.

### iPLCs contraction assay

The assay protocol was adapted from Neuhaus et al., 2017 [26] and Hibbs et al., 2021 [27] with some modifications. Before seeding the cells, 50 µl of the medium was applied into impedance plates (E-Plate 16, Agilent) coated with 100-fold diluted Matrigel for baseline recording. Subsequently, iPLCs were re-plated onto the plate at a density of 6.25 × 10^4^/cm^2^ and allowed to adhere for 30 min at RT. Cell attachment was monitored by recording the cell index at 15-min intervals using xCELLigence® RTCA (Agilent) until cells reached the confluence before the assay, which typically takes 16 to 30 h. Endothelin-1 (ET-1, Merck) was then introduced to the culture at a final concentration of 10 nM and the cell index was recorded at 15-s intervals for 2.5 h and followed by 15-min intervals for the subsequent 27.5 h. Since ET-1 was reconstituted in water, an additional 0.1% of water (final concentration) in the medium served as vehicle control. The ‘cell index’ was calculated by software using the cell layer`s resistance to electrical current, which reflected the contact area between cells and the well surface. The baseline-normalized cell index was generated by normalizing the raw cell index against the mean index of duplicate vehicle controls and the index at the time point when ET-1 is applied to the well. Data analysis was performed with RTCA Software Pro (Agilent).

### Cytokines production detection

The Cytometric Bead Array with human soluble protein flex sets (BD Biosciences) was used to examine cytokines secreted by iPLCs. The medium was collected from wells pre-stimulated with 20 ng/mL tumor necrosis factor (TNF) α and 20 ng/mL interleukin (IL)-1β (both from PeproTech, Thermo Fisher Scientific) for 24 h. As another stimulant, we tried a combination of 100 ng/ml lipopolysaccharide (LPS; Merck) and 15 ng/ml interferon γ (IFNγ; PeproTech), which acts as a powerful stimulant on iPSC-derived microglia in our hands [28]. Conditioned medium was diluted 10 times with assay diluent for TNFα and IL-1β-stimulated samples. 20 µl of samples or standards were incubated with 20 µl beads mixture (75 times dilution) for 1 h at RT, followed by 2 h of incubation after adding 20 µl detection reagent (75 times dilution). Samples were run on BD Accuri™ C6 Flow Cytometer (BD Biosciences) by detecting around 200 to 300 events for each cytokine. Beads were clustered with 675/25 nm (APC) and 780/60 nm (APC-Cy7) optical filters, and cytokines were quantified using 585/40 nm (PE) filter. The data were analyzed with the FCAP array (SoftFlow), and the absolute concentration of secreted cytokines was calculated according to each cytokine’s standard regression curve.

### RNA-sequencing and analysis

iPLCs samples for RNA sequencing (RNA-seq) were harvested on day 21 after differentiation started. The density of cells was evaluated from identical wells ranging from 0.8 to 1.6 × 10^6^ cells per well in 6-well plates to ensure cell confluency was comparable between the lines. Total RNA was extracted using the RNeasy Mini Kit following the manufacturer’s manual. DNase (Molecular grade; Thermo Fisher Scientific) was introduced during the RNA isolation procedure to produce DNA-free samples following manufacturer’s manual. RNase inhibitor RiboLock was added after the elusion step to inhibit RNase activity for better RNA preservation. The RNA quantity and quality were analyzed with TapeStation 4200 (Agilent). The Illumina Stranded Total RNA Prep with Ribo-Zero Plus kit (Illumina) was used to deplete ribosomal RNA and prepare RNA-seq libraries. The libraries were then sequenced with NextSeq500 (Illumina). The total number of reads was over 30 million per sample. The library preparation and sequencing service were provided by the Biomedicum Functional Genomics Unit (FuGu) at the Helsinki Institute of Life Science and Biocenter Finland at the University of Helsinki. For detection of differentially expressed genes (DEG), raw sequence reads were aligned to human genome GRCh.38 and annotated to gene exons by STAR aligner v2.7.8a [29] and HTSeq v0.13.5 [30] with GTF v103, respectively. DEG analysis of APPswe pericytes against healthy control samples were performed using the DESeq2 package in R [31]. With the cutoff settings (FDR < 0.05 and absolute log2 fold change > 1.5), clustering heatmaps were created with R package heatmap 1.0.12 to show DEGs. In addition, using the same cutoff values, pathway enrichment analyses were done by knowledge-based Ingenuity Pathway Analysis (IPA; Qiagen).

### Statistics

Statistical analyses were performed using GraphPad Prism 5.01 and 9.3.1 software (GraphPad Software Inc). Columns were compared with Student’s t‐test or one‐way ANOVA with Dunnett’s multiple comparison test. Groups with two variables were analyzed by two‐way ANOVA with Bonferroni multiple comparison test. Statistical significance was determined with the p-value < 0.05. All data in the graphs are shown as mean ± SD.

## Results

### iPSCs efficiently differentiate into iPLCs

Pericytes were differentiated from human iPSCs (Table 1) via mesodermal route according to a slightly modified protocol by Blanchard and coworkers [22]. Given the lack of markers that can reliably differentiate between pericytes and SMCs, we refer to the generated cells as iPSC-pericyte-like cells (iPLCs) in our study.

**Fig. 1:**
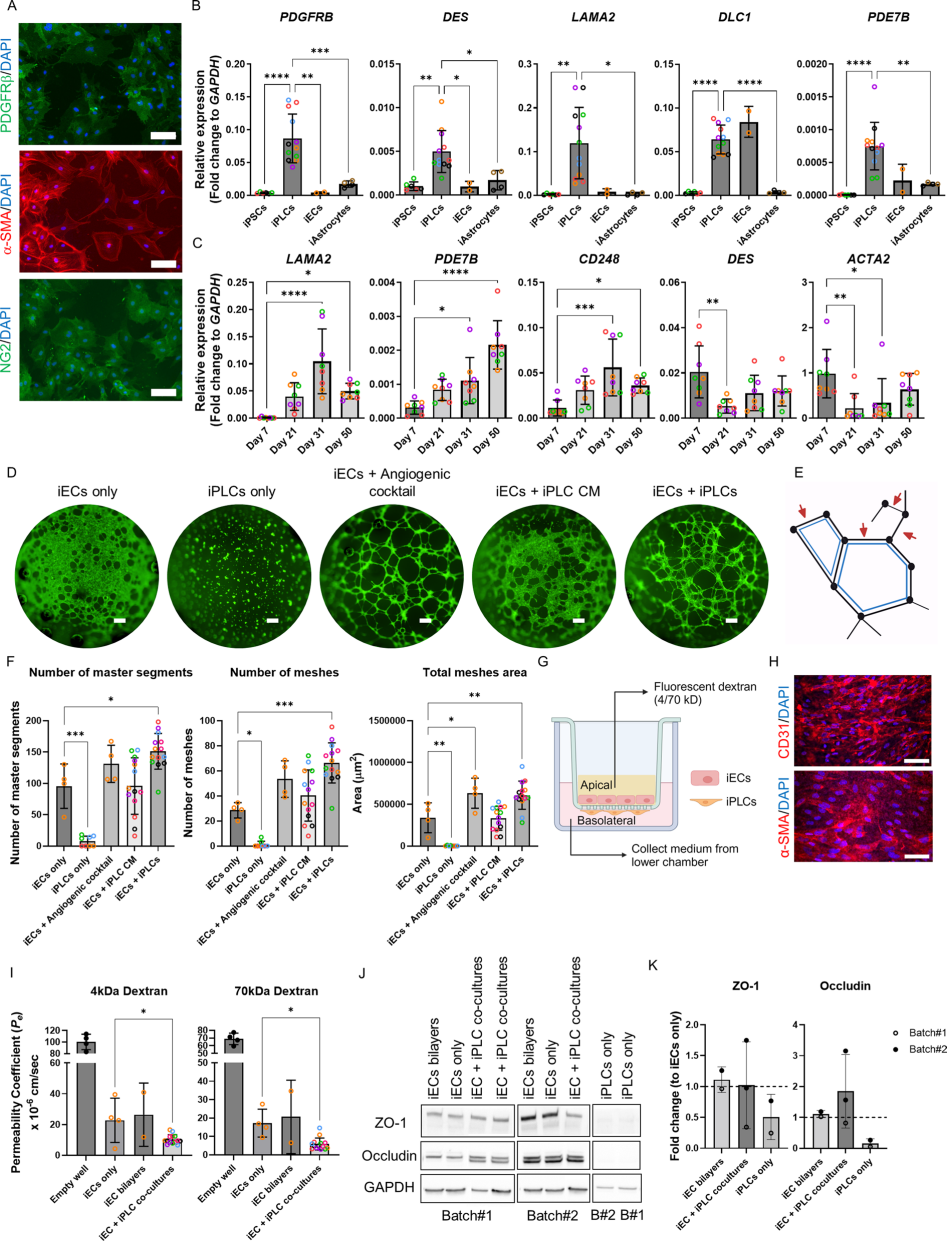
Differentiation and characterization of iPSC-derived pericyte-like cells (iPLCs). **A** Immunostaining for PDGFRβ, α-SMA, and NG2 in day 21 iPLCs derived from control lines. Nuclei are stained with DAPI. Scale bars, 100 μm. **B** Comparison of relative gene expression levels for *PDGFRB*, *DES*, *LAMA2*, *DLC1*, and *PDE7B* among iPLCs, iECs, iAstrocytes, and iPSCs. Expression levels are shown as fold change relative to *GAPDH*. **C** Relative gene expression levels of *LAMA2*, *PDE7B*, *CD248*, *DES*, and *ACTA2* in iPLCs across Day 7, 21, 31, and 50. Expression levels are shown as fold change relative to *GAPDH*. **D** 2D tube formation images showing iECs alone, iPLCs alone, iECs exposed to angiogenic cocktail or iPLC-conditioned medium (CM), and iECs co-cultured with iPLCs. Scale bars, 300 μm. **E** Illustration of master segments (red arrows) and mesh structures (blue polygon). **F** Statistical analysis of the number of master segments, meshes count, and meshes area. **G** Schematic of the experimental setup for permeability assays. **H** Immunostaining of CD31 and α-SMA on iECs and iPLCs cultured on Transwell inserts. Nuclei are stained with DAPI. Scale bars, 100 μm. **I** Endothelial permeability coefficients (*Pe*) for 4 kDa and 70 kDa fluorescently labeled dextran across iEC only, iEC bilayers, iECs in co-culture with iPLCs cultures and empty well without cells on inserts as control. **J–K** Western blots for ZO-1 and Occludin from two experimental batches, analyzing iEC-only, iPLC-only, iEC bilayers, and iECs in co-culture with iPLCs. GAPDH served as a loading control (**J**). Statistical analysis depicting fold changes in ZO-1 and Occludin expression levels relative to iEC-only controls for each batch (**K**). The dots indicate the average values of technical replicates for each biological sample (lines, batches), with the color of the dots representing different lines. Except in (**I**), where the dots from empty well represent the data from one well of one experimental batch. The data are presented as mean ± SD. Statistical analysis utilized one-way ANOVA with Dunnett's multiple comparison test, with significance denoted: *p < 0.05, **p < 0.01,***p < 0.001 and ****p < 0.0001

Our iPLCs exhibited positive immunoreactivity for mural cell markers platelet-derived growth factor receptor beta (PDGFRβ), neural-glial antigen 2, proteoglycan (NG2), and alpha-smooth muscle actin (α-SMA) (Fig. 1A). Additionally, these cells displayed significantly higher expression levels of mural cell-enriched genes [9] *PDGFRB*, *DES*, *LAMA2*, and *PDE7B,* in comparison to undifferentiated iPSCs, iECs, and iAstrocytes) (Fig. 1B). While *DLC1* expression was also elevated in iECs, iPLCs still exhibited a significantly higher expression level than both iPSCs and iAstrocytes. As expected, the cells did not express the pluripotency markers *NANOG*, *LIN28A*, and *SOX2*, nor the endothelial cell marker *CDH5* (Figure S2 A-B). To determine the best timeline to conduct our experiments, we examined the expression levels of pericyte-associated genes in four control lines at days 7, 21, 31, and 50 after the initiation of differentiation. We discovered a gradual rise in the expression of *LAMA2, PDE7B,* and another mural cell marker *CD248* until day 31 of the culture period (Fig. 1C*).* The expression of *PDE7B* increased even further on day 50. In contrast, the expression of SMC-associated genes *DES* and *ACTA2* decreased significantly from day 7 to day 21 (Fig. 1C). *PDGFRB* and *DLC1* gene expression levels remained stable across the analyzed time points (Figure S2 C). These results imply that the cells acquired a pericyte-like identity between day 21 and 31 post-differentiation. To minimize the variation, we chose the time-point of 21–23 days post-differentiation for further experiments.

### iPLCs promote angiogenesis and barrier integrity

The regulation of vascular function and morphology depends on the interactions between endothelial cells and vascular mural cells. To explore the angiogenic potential of iPLCs, a 2D tube formation assay was performed. For this purpose, iECs were derived from the Ctrl2 line transduced with ETV2 under the doxycycline-inducible promoter. The expression of ETV2 was induced on day three after the initiation of differentiation. Initially, we evaluated the angiogenic capabilities of iECs and iPLCs independently, finding that neither could form clear tube-like structures on a Matrigel layer when cultured alone (Fig. 1 D). The analysis was conducted using the ImageJ Angiogenesis Analyzer, which converts images to binary format and identifies tube-like structures. We used parameters such as segment number, mesh number, and area (Fig. 1E) to determine the complexity of the tube structures. Due to converting pixels to binary, even without visible segments or meshes in iECs or iPLCs cultures, the binary images can still produce pseudo structures with low number counts. Subsequently, we examined the ability of iECs to form tubes when subjected to angiogenic factors. Notably, iECs exposed to an angiogenic cocktail (3 ng/mL VEGF-A, 30 nM sphingosine-1-phosphate (S1P), 3 ng/mL phorbol 12-myristate 13-acetate (PMA) and 2.3 ng/mL bFGF) successfully formed complex tubular structures, with a significant increase in the total mesh area (Fig. 1D, F). The total numbers of master segments and meshes also tended to be increased following the stimulation with the angiogenic cocktail (Fig. 1F). iPLCs-conditioned medium (CM) did not significantly induce tube formation in iECs. However, direct co-culturing of iECs with iPLCs resulted in a significant increase in the numbers of master segments and meshes as well as the total mesh area (Fig. 1D, F). These observations suggest that iPLCs may play a crucial role in promoting vessel formation and stabilization by directly interacting with iECs.

Additionally, the ability of iPLCs to induce BBB properties in iECs was evaluated using a Transwell model. In the experimental setup, iPLCs were seeded on the basolateral side of the inserts, while iECs were cultured on the apical side (Fig. 1G). As controls, iECs grown alone (iECs only) and iECs plated on both apical and basolateral sides (iEC bilayers) were used. Initially, CD31 and α-SMA stainings were conducted to confirm the proper attachment of iECs and iPLCs to the membrane before performing the permeability assay (Fig. 1H). The permeability to 4 kDa and 70 kDa dextran of 7-day-old co-cultures was significantly reduced (Fig. 1I) compared to apical iECs alone. The Western blot analysis showed that the presence of iPLCs did not significantly affect RIPA-soluble levels of the tight junction proteins (TJPs) ZO-1 and occludin as compared to iECs alone (Fig. 1 J–K). These findings indicate that iPLCs regulate the formation and integrity of blood vessels, thereby confirming their functional efficacy.

### APPswe iPLCs exhibited differential expression of pericyte markers and expressed α-SMA stress fibers

**Fig. 2:**
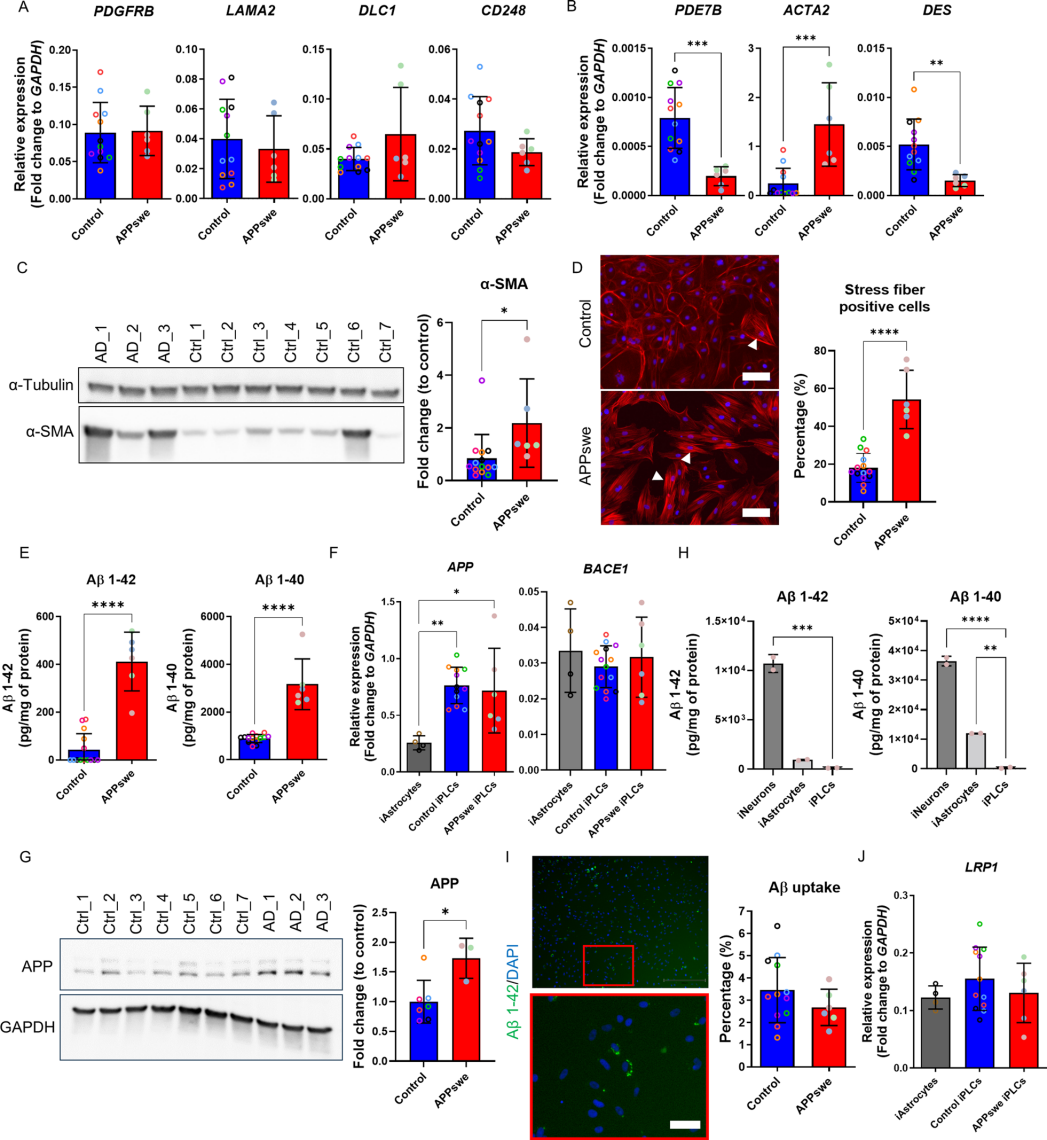
APPswe iPLCs displayed altered expression of pericyte markers, α-SMA stress fibers, and amyloid beta pathology. **A**–**B** Comparative analysis of relative expression levels of *PDGFRB*, *LAMA2*, *DLC1*, *CD248* (**A**), and *PDE7B*, *ACTA2*, *DES* **B** between control and APPswe iPLCs. Expression levels are shown as fold change relative to *GAPDH*. **C** Representative blots for α-SMA in control and APPswe iPLCs, using α-tubulin as a loading control. **D** Immunostaining of α-SMA in control and APPswe iPLCs, with nuclei stained by DAPI. Scale bars, 100 μm. The percentage of cells with stress fibers was quantified using ImageJ's threshold function to measure cell coverage. **E** Aβ1–42 and Aβ1–40 levels in media from control and APPswe iPLCs, normalized to total protein content. **F** Relative gene expression levels of *APP* and *BACE1* in control and APPswe iPLCs, as well as iAstrocytes, quantified as fold changes relative to *GAPDH*. **G** Western blot for APP in control and APPswe iPLCs,with GAPDH as the loading control. **H** Aβ1–42 and Aβ1–40 levels measured in media from iPSC-neurons, astrocytes, and iPLCs derived from an APPswe individual. The obtained values were normalized to total protein content. **I** Images of iPLCs internalizing HiLyte 488-labeled Aβ1–42, displayed at 4 × and 10 × magnifications, scale bar 100 μm (10x). The percentage of cells internalizing Aβ1–42 was quantified using 10 × images. **J** Relative gene expression levels of *LRP1* in control iPLCs, APPswe iPLCs, and iAstrocytes, quantified as fold changes relative to *GAPDH*. The dots indicate the average values of technical replicates for each biological sample (lines, batches), with the color of the dots representing different lines. The data are presented as mean ± SD. Statistical analysis was performed using one-way ANOVA with Dunnett’s multiple comparison test (F, H, J) or t-test (A, B, C, D, E, G, I). The significance levels are denoted as follows: *p < 0.05, **p < 0.01,***p < 0.001 and ****p < 0.0001

We expanded our investigation to compare the expression of pericyte genes between iPLCs derived from healthy individuals and those carrying APPswe mutation. Our analysis revealed no significant differences in the expression levels of *PDGFRB*, *LAMA2*, *DLC1*, and *CD248* between APPswe and control iPLCs (Fig. 2A). However, a notable reduction in the expression of *PDE7B* and *DES* was observed in APPswe iPLCs, alongside an increase in *ACTA2* expression (Fig. 2B). *ACTA2* encodes for α-SMA, a protein associated with cell contractility, and α-SMA protein levels were also significantly elevated in APPswe iPLCs (Fig. 2C). A detailed analysis of α-SMA staining revealed distinct organizational patterns. In control iPLCs, α-SMA was diffusely distributed and primarily located at the cell margins. In contrast, in APPswe iPLCs, there was a noticeable increase in the presence of actin stress fibers (Fig. 2D). The quantification of cells exhibiting actin stress fibers revealed a significant increase in the prevalence of these structures among iPLCs with APPswe mutation (Fig. 2D). These findings suggest potential defects in APPswe iPLCs.

### iPLCs can produce and secrete Aβ peptides

The accumulation of Aβ deposits in the brain is a hallmark of AD. Previous research has reported the co-labeling of pericytes with Aβ in human AD brain [32, 33]. However, it is not clear whether pericytes actively produce Aβ or uptake it from the surrounding extracellular space. We found that iPLCs harboring APPswe mutation secreted significantly more Aβ1-42 (413.7 ± 88.4 pg/mg cell lysate over 7 days; Fig. 2E) compared to control cells (43.4 ± 54.7 pg/mg cell lysate; Fig. 2E). As expected, iPLCs secreted higher levels of Aβ1-40 than Aβ1-42 (Controls: 894.2 ± 125.8 pg/mg; APPswe: 3165.4 ± 701.7 pg/mg), and the genotype effect was similar to the effect on Aβ1-42 (Fig. 2E). The mRNA level of *BACE1* in both control and APPswe iPLCs was similar to those found in iAstrocytes (Fig. 2F), while the level of *APP* was significantly higher in iPLCs. Notably, APP protein levels were higher in APPswe iPLCs compared to controls (Fig. 2G). Our data suggest that pericytes can produce Aβ and may contribute to the Aβ pathology, although the extent of their contribution is yet to be fully understood. To clarify this, we compared Aβ production by iNeurons, iAstrocytes, and iPLCs to gauge the relative contribution of pericytes to the overall Aβ pathology. We found that APPswe iPLCs secreted on average 100 times lower levels of both Aβ1-42 and Aβ1-40 than APPswe iNeurons when grown at the same cell density for 72 h (Fig. 2H). The findings suggest that the contribution of pericytes to total brain amyloid load in AD is limited.

To investigate Aβ uptake potential of our iPLCs, we introduced fluorescently labeled fibrillar Aβ1-42 to the cultures and incubated them for 24 h. Only around 3% of control cells ingested Aβ1-42, and the APPswe mutation did not affect Aβ1-42 uptake (Fig. 2I). Both control and APPswe iPLCs expressed *LRP1* mRNA levels similar to iAstrocytes, known to express detectable levels of *LRP1* [34] (Fig. 2J). Furthermore, when iPLCs were subjected to pHrodo-conjugated zymosan-coated beads, no uptake of these pathogen-mimicking particles was observed. Thus, it appears that the phagocytic activity of these iPLCs is low.

### APPswe mutation significantly altered the transcriptome of iPLCs

**Fig. 3:**
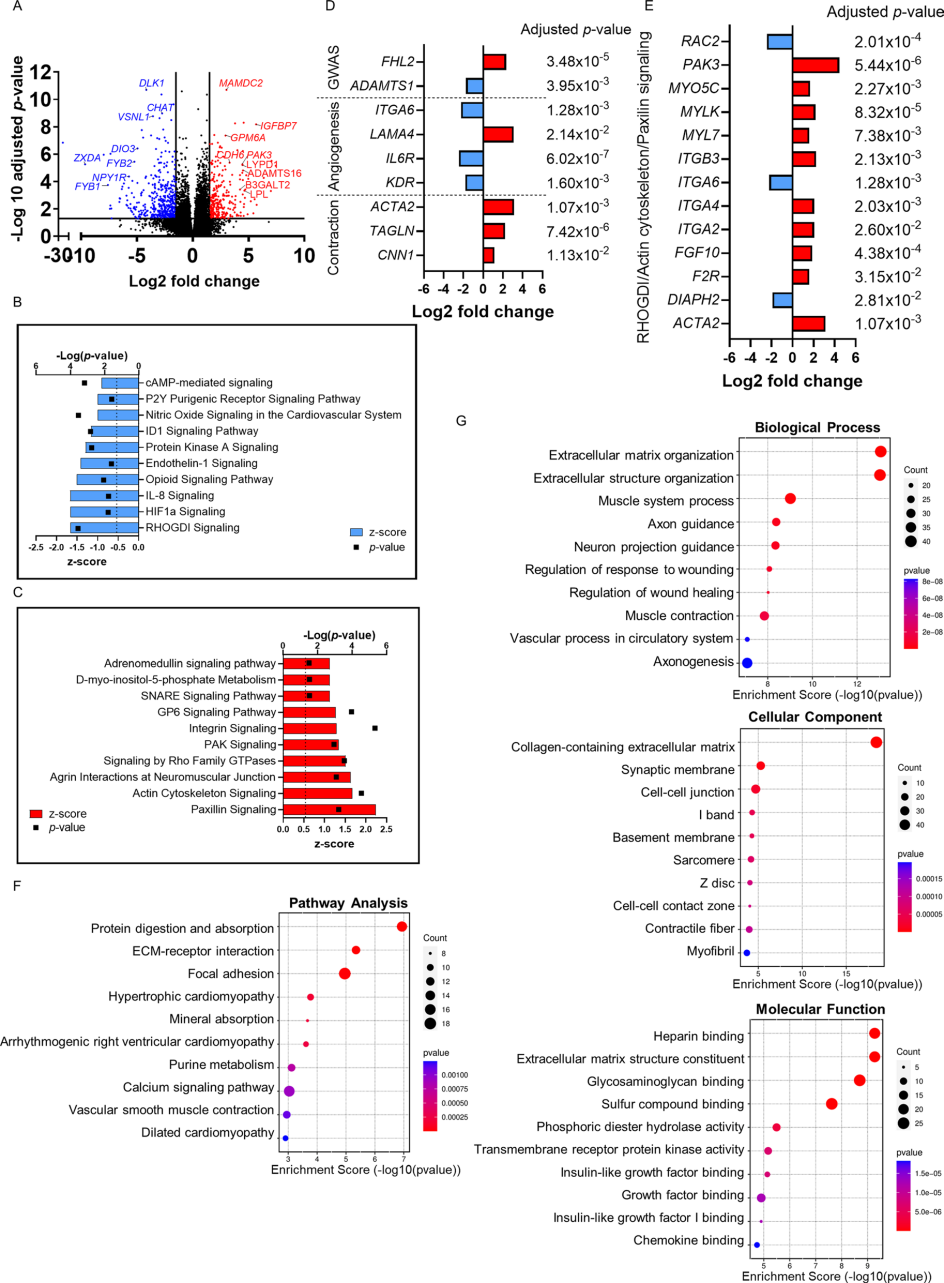
APPswe iPLCs exhibit an altered transcriptome. **A** Volcano plot depicting DEGs between control and APPswe iPLCs (cutoffs: Adjusted *p*‐value < 0.05 and an absolute log2 fold change > 1.5). The analysis included seven control and three APPswe lines. **B**–**C** Ingenuity Pathway Analysis identifying the top 10 canonical pathways that are most significantly downregulated (**B**) and upregulated (C) in APPswe iPLCs compared to controls, with pathways selected based on the highest z-scores (*p*-value < 0.05). **D** List of DEGs related to AD risk from GWAS, angiogenesis and pericyte contraction process (cutoffs: Adjusted *p*‐value < 0.05 and an absolute log2 fold change > 1). **E** List of genes involved in RHOGDI, Paxillin and Actin Cytoskeleton signaling pathways. **F** Pathview pathway analysis identifying the top 10 pathways most affected in APPswe iPLCs relative to controls, ranked by the highest enrichment scores. **G** Gene ontology (GO) enrichment analysis revealed pathways enriched in Biological Process, Molecular Function, and Cellular Component in APPswe iPLCs relative to controls

To further investigate the impact of the APPswe mutation on iPLCs, we conducted transcriptomic analysis on 21-day-old cells. A pairwise comparison identified 687 differentially expressed genes (DEGs) between APPswe and control iPLCs (Figure S3 A, Table S2), with 257 genes upregulated and 430 downregulated. The topmost up- and downregulated genes in APPswe iPLCs are highlighted in red and blue in volcano plot, respectively (Fig. 3A). We used Ingenuity Pathway Analysis (IPA) to identify the pathways most affected by APPswe mutation in iPLCs (Fig. 3B, C, Table S3). Among the five strongest downregulated pathways, we identified Rho GDP dissociation inhibitor (RHOGDI) signaling, hypoxia inducible factor (HIF)1α signaling, IL-8 signaling, opioid signaling, and ET-1 signaling (Fig. 3B). The ET-1 pathway regulates vasoconstriction [35] while IL-8 and HIF1α signaling pathways are involved in angiogenesis, inflammation, and metabolic regulation [36, 37]. The top five upregulated pathways in APPswe iPLCs were associated with actin/cytoskeleton reorganization, including paxillin signaling, actin cytoskeleton signaling, agrin interactions at neuromuscular junction, signaling by RHO family GTPases, and PAK signaling (Fig. 3C). Among the DEGs shared by these pathways, there was a significant number of genes related to myosin chains, smooth muscle actin, and integrins (Fig. 3D, E). Additionally, the DEGs involved in the regulation of angiogenesis included *IL6R* and *KDR (*Fig. 3D). Another pathway enrichment analysis using Pathview revealed significant changes in vascular smooth muscle contraction and cardiomyopathy-related pathways, extracellular-receptor interaction and focal adhesion pathways (Fig. 3F). Detailed examination of genes within these pathways identified *ITGA2*, *ITGA4*, *ITGA6*, and *MYLK*, which are involved in cytoskeleton reorganization as described in IPA analysis (Table S4). Gene ontology analysis further underscored an enrichment in biological processes associated with vascular and muscle functions and extracellular matrix/structure organization (Fig. 3G, Tables S5-7), which are crucial for vascular stability and pericyte fate determination [38]. This analysis also emphasized an enrichment in cellular components of contractile fibers and myofibers (Fig. 3G, Tables S5-7).

In summary, our transcriptomics data suggests that APPswe iPLCs may exhibit altered functionalities such as cell contractility, inflammatory response, and metabolism regulation.

### APPswe iPLCs produce higher levels of MCP-1 after inflammatory stimulation

**Fig. 4:**
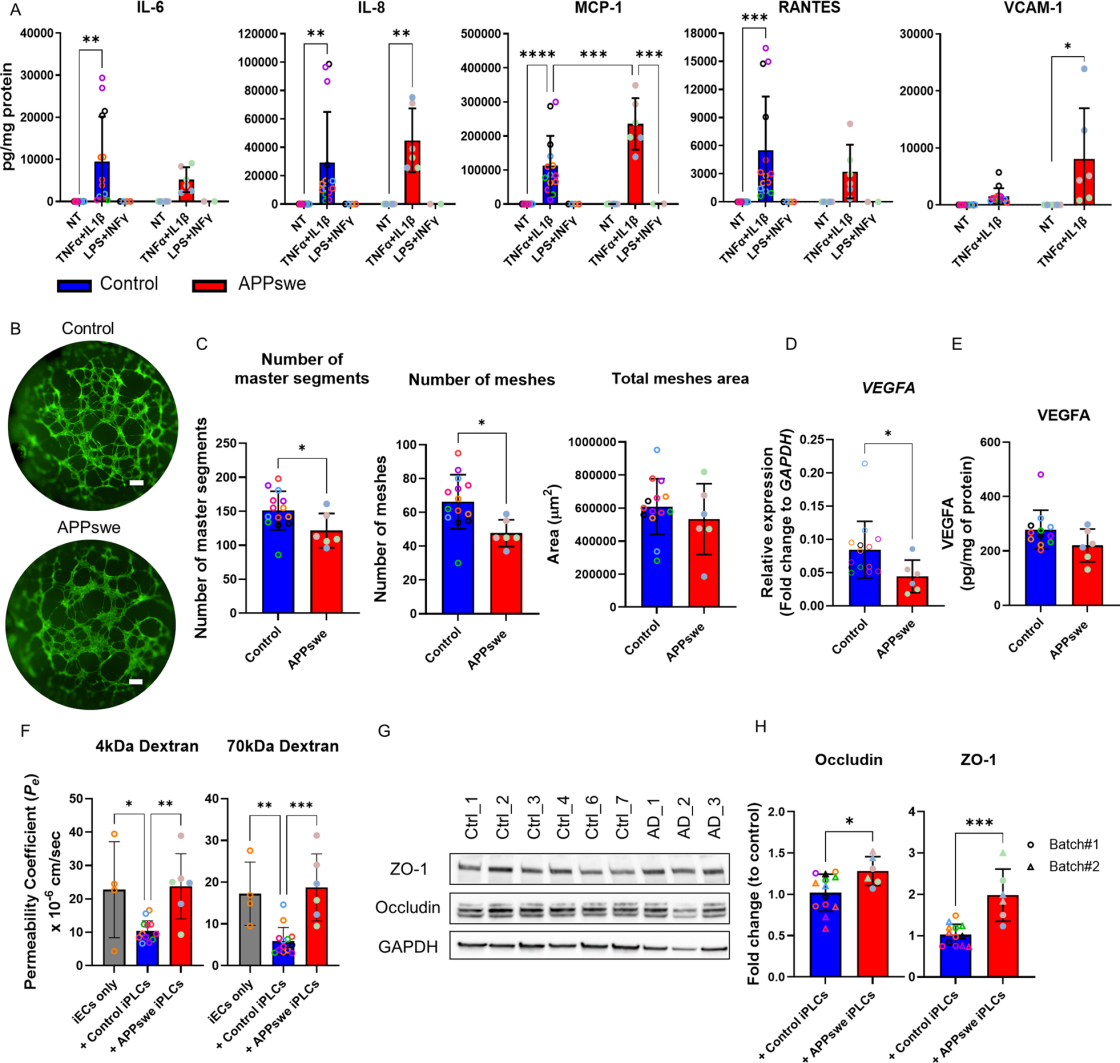
APPswe iPLCs show functional deficits in responding to inflammation, supporting angiogenesis, and maintaining barrier integrity. **A** IL-6, IL-8, MCP-1, RANTES, and VCAM-1 concentrations were measured in iPLCs culture media after 24 h of stimulation with TNFα and IL-1β or LPS and IFNγ stimulation. Results were normalized to total protein content. Statistical differences between treatments and genotypes were indicated. **B**–**C** 2D tube formation assays involved culturing iECs alongside both control and APPswe iPLCs (**B**), scale bars, 300 μm. Statistical comparisons were made regarding the number of master segments, number of meshes, and mesh area between co-cultures of control and APPswe iPLCs with iECs (**C**). **D** Relative gene expression levels of *VEGFA* in control and APPswe iPLCs, quantified as fold changes relative to *GAPDH*. **E** VEGF-A protein levels in lysates from control and APPswe iPLCs were measured and normalized to total protein content. **F** *Pe* for 4 kDa and 70 kDa fluorescently labeled dextran, assessed in iECs only cultures and iECs in co-culture with either control or APPswe iPLCs. **G** Representative blots of ZO-1 and Occludin from control and APPswe iPLCs, with GAPDH as the loading control. **H** Quantification results from two batches. The dots indicate the average values of technical replicates for each biological sample (lines, batches), with the color of the dots representing different lines. The data are presented as mean ± SD. Statistical analysis was performed using two-way ANOVA with Bonferroni multiple comparison test (**A**), one-way ANOVA with Dunnett’s multiple comparison test (F) or t-test (**C**–**E, H**). The significance levels are denoted: *p < 0.05, **p < 0.01,***p < 0.001 and ****p < 0.0001

Pericytes can sense inflammatory stimuli and activate innate immune responses, such as the release of pro-inflammatory cytokines and overexpression of adhesion molecules, including intercellular adhesion molecule (ICAM)-1 and vascular cell adhesion molecule (VCAM)-1 [39, 40]. Given that our transcriptome analysis (Fig. 3D) showed a downregulation of IL-8 signaling pathway in APPswe iPLCs, we tested the effect of a 24 h exposure to a combination of proinflammatory cytokines TNFα and IL-1β (Fig. 4A) on these cells. Following the exposure, inflammatory mediators IL-6 (two-way ANOVA, *p* = 0.0427), IL-8 (*p* = 0.0028), monocyte chemoattractant protein (MCP)-1 (CCL2) (*p* < 0.0001), regulated on activation, normal T cell expressed and secreted (RANTES; CCL5) (*p* = 0.0267), and soluble VCAM-1 (*p* = 0.0156), were secreted by iPLCs. It is worth noting that bacterial lipopolysaccharide failed to induce this response (Fig. 4 A), potentially due to extremely low expression levels of Toll-like receptor (*TLR) 2* and *TLR4* as well as *CD14* genes (Table S8). Upon stimulation with TNFα and IL-1β, APPswe iPLCs secreted significantly higher levels of MCP-1 and showed a rising trend in soluble VCAM-1 (*p* = 0.0517) compared to control cells (Fig. 4A). No significant genotype effect was observed on the levels of the remaining inflammatory mediators tested. This suggests that APPswe iPLCs are sensitive to inflammatory stimuli, resulting in an increased release of certain pro-inflammatory mediators.

### APPswe iPLCs impair angiogenesis and barrier integrity

Given the observed changes in angiogenesis-related gene expression within the APPswe iPLCs transcriptome compared to controls (Fig. 3B), we used tube formation and dextran permeability assays to evaluate the ability of APPswe iPLCs to support normal endothelial cell functions. In co-cultures of iECs with APPswe iPLCs, the network of self-assembled tubes was less complex compared to those formed with control iPLCs (Fig. 4B). Statistical analysis revealed a significant reduction in the number of segments and meshes in iECs co-cultured with APPswe iPLCs relative to control cells (Fig. 4C). However, no differences were observed in the total mesh area. These results suggest that APPswe iPLCs have a diminished capacity to support angiogenesis. Since VEGF-A is the major stimulator of angiogenesis [41, 42] and *VEGFA* mRNA levels tended to be lower in the transcriptomic dataset (Table S8), we checked the levels of *VEGFA* expression by RT-qPCR (Fig. 4D). Consistent with RNA-seq data, *VEGFA* mRNA levels were reduced in APPswe iPLCs compared to the control group. We then checked the VEGF-A protein levels by ELISA. However, no difference was observed in VEGF-A levels in cell lysates between APPswe and control iPLCs (Fig. 4E), and the secretion of soluble VEGF-A into the culture medium was undetectable for both groups.

Consistent with previous findings, the permeability of iECs to both 4 kDa and 70 kDa fluorescently labeled dextran was notably reduced in co-cultures with control iPLCs, as opposed to iEC-only cultures and co-cultures with iECs on the basolateral side (Fig. 4F). However, permeability significantly increased when iECs were co-cultured with APPswe iPLCs compared to those with control cells, aligning with iEC-only cultures (Fig. 4F). This observation indicates that APPswe iPLCs undermine the integrity of the endothelial barrier. To check whether the degradation of the TJPs could explain these differences, we conducted a Western blot analysis. Surprisingly, the levels of RIPA-soluble ZO-1 and occludin were increased in APPswe pericytes when normalized to GAPDH (Fig. 4G, H), indicating that our results could not be explained by TJP degradation and that further research is needed to understand the underlying mechanisms.

### APPswe iPLCs exhibit a prolonged response to ET-1 treatment

Since we had detected a higher prevalence of stress fibers (Fig. 2D) and upregulation of cytoskeleton reorganization pathways in APPswe iPLCs (Fig. 3E–G), we aimed to further investigate potential defects in pericyte contractility. To validate the contractile ability of iPLCs in response to vasoconstricting and vasodilating signals, we exposed our cultures to ET-1 or adenosine triphosphate (ATP), respectively. We then monitored cell contraction process using the xCELLigence system.

**Fig. 5:**
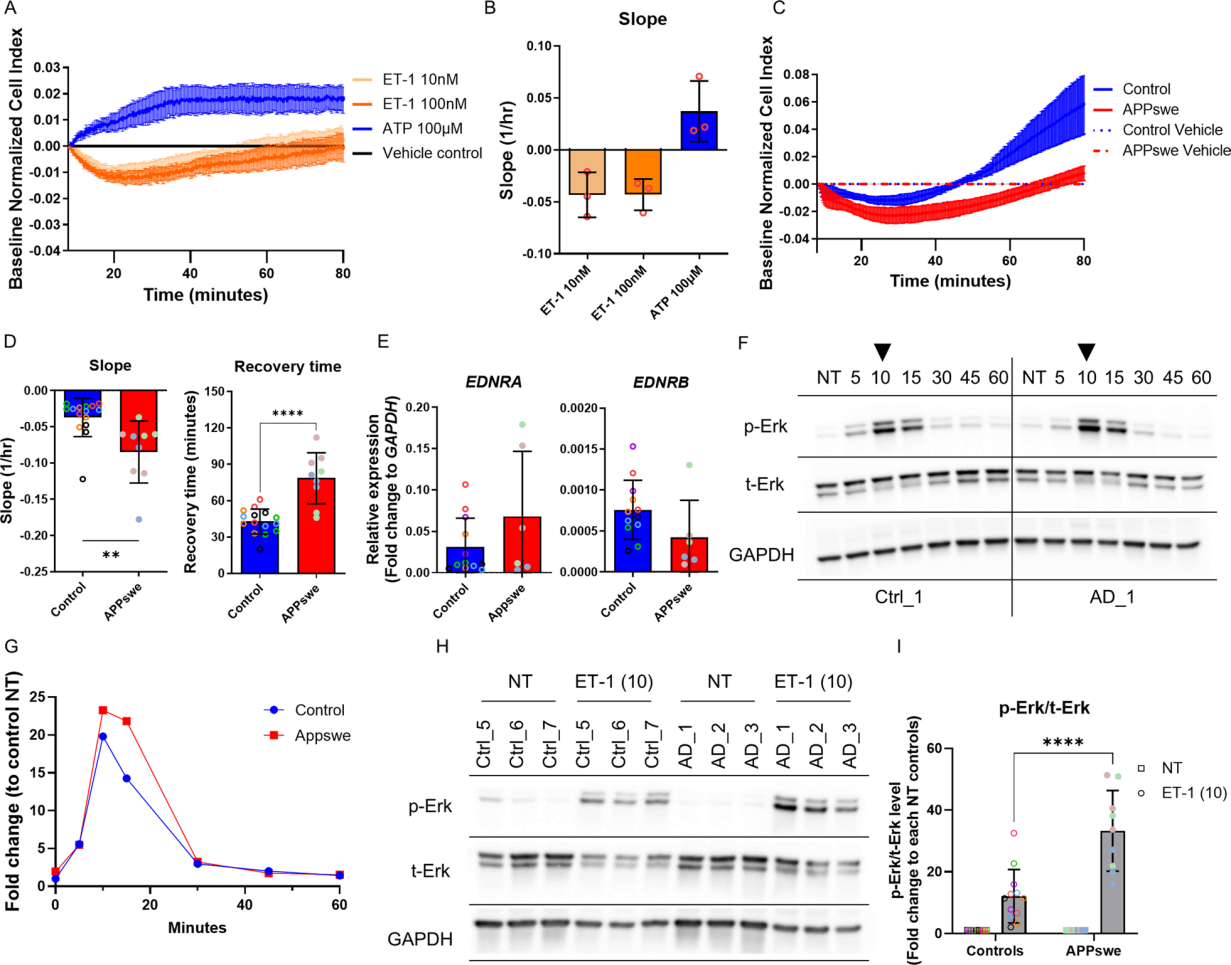
APPswe iPLCs exhibited a hypercontractile phenotype. **A–B** Electrical impedance measurements evaluated the contractile response of iPLCs to various concentrations of ET-1 and ATP. Response curves, normalized to vehicle control, depicted cell index showing the response (**A**), while the response slope indicated contraction speed post-treatment (**B**). (**C**) Cell index of the contractile response to ET-1 treatment between control and APPswe iPLCs. **D** Statistical analysis of response slope and recovery time for iPLCs returning to normal size. **E** Gene expression levels of *EDNRA* and *EDNRB* in control versus APPswe iPLCs, quantified as fold changes relative to *GAPDH*. **F–G** Time-resolved blots for phosphorylated (p)-Erk and total (t)-Erk in control and APPswe iPLCs post-ET-1 treatment at intervals of 5, 10, 15, 30, 45, and 60 min, as well as in non-treated (NT) cells (**F**), with quantifications normalized to GAPDH and control NT wells (**G**). (**H**–**I**) Blots of p-Erk and t-Erk from control and APPswe iPLCs 10 min after ET-1 treatment and from NT cells, with GAPDH serving as the loading control (**H**). Blot quantification was normalized to GAPDH levels, and treatment groups were further normalized to the NT wells of their respective lines (**I**). The dots indicate the average values of technical replicates for each biological sample (lines, batches), with the color of the dots representing different lines. The data are presented as mean ± SD. Statistical analysis was performed using two-way ANOVA with Bonferroni multiple comparison test (**I**) or t-test (**D**, **E**). The significance levels are denoted: *p < 0.05, **p < 0.01,***p < 0.001 and ****p < 0.0001

This system measures the electric impedance of the cell layer converting it into a cell index that reflects changes in the cell surface area. The iPLCs exhibited immediate contraction in response to ET-1 administration, as shown by a reduction in cell index (Fig. 5A). After the initial contraction, there was an increase in cell index 20–30 min post-ET-1 treatment, suggesting cell relaxation. In contrast, when ATP was used as a vasodilator, it caused an elevation in the cell index (Fig. 5A), indicating a potential relaxation of the cells. Further analysis of the contraction/relaxation dynamics, as inferred from the slope changes, showed no significant differences between cells exposed to 10 nM and 100 nM of ET-1 (Fig. 5B). Consequently, we chose 10 nM ET-1 for subsequent experiments.

After confirming basic contractile responses, we investigated the behavior of both control and APPswe iPLCs. Compared to the control group, APPswe iPLCs exhibited a steeper contraction slope (Fig. 5C, D). They also required a significantly prolonged recovery period to attain normal cell index (Fig. 5C,D). Next, we assessed the transcriptomics data to determine the expression levels of ET-1 receptors. The iPLCs expressed both *EDNRA* and *EDNRB* genes, but the former was expressed on average 10 times higher than the latter (Table S8)*.* This suggests that endothelin receptor type A may be the primary ET-1 receptor in iPLCs. Nonetheless, there were no significant differences in the expression of these genes between the genotypes. These results were confirmed using RT-qPCR (Fig. 5E). Since ET-1 receptors are G-protein-coupled receptors and extracellular signal-regulated kinase 1 and 2 (Erk1/2) is a common downstream pathway, we collected cells at various time points following ET-1 treatment to observe alterations in downstream signaling. The levels of Erk phosphorylated at Thr202 and Tyr204 (p-Erk) peaked in both control and APPswe iPLCs around 10 min post-treatment, with a decline thereafter (Fig. 5F). Interestingly, the p-Erk/t-Erk ratio at 10 min post-ET-1 treatment increased more in APPswe iPLCs than in controls (Fig. 5H–I). These observations suggest that APPswe iPLCs exhibit a hypercontractile phenotype with prolonged recovery post-ET-1 exposure. However, further research is needed to confirm the significance of the elevated p-Erk/t-Erk ratio.

## Discussion

In this study, we generated human iPLCs carrying a genetic variant linked to familial AD. These iPLCs expressed high levels of common pericyte marker genes. Interestingly, some pericyte marker genes such as *PDE7B*, *ACTA2*, and *DES* exhibited differential expression in APPswe iPLCs compared to controls. These changes are more likely caused by intrinsic properties of the APPswe mutation in iPLCs rather than differentiation guidance failures.

A key discovery of our study is a novel mechanism potentially contributing to CAA pathology. Previous studies reported Aβ production by cerebrovascular cells [43, 44]. However, the significance of these findings has remained ambiguous because Aβ accumulation in the vasculature is generally thought to originate predominantly from neurons [45]. Indeed, we see that iPLCs produce substantially lower levels of Aβ than neurons. However, our findings reveal that APPswe iPLCs produced on average 10 times higher levels of Aβ1-42 and 3.5-fold higher levels of Aβ1-40 as compared to controls. Given their localization, pericytes may still contribute to vascular amyloidosis.

We also show that APPswe iPLCs have an impaired capacity to support vessel formation, stabilization, and barrier integrity, exhibit a prolonged contractile response, and produce increased levels of pro-inflammatory cytokines upon inflammation.

The hypercontractile phenotype of APPswe iPLCs was accompanied by an increased mRNA expression of *ACTA2*, which encodes for α-SMA, and other cytoskeleton-regulating proteins. In SMCs, the contractile functions are primarily dependent on contractile proteins such as α-SMA, smooth muscle myosin heavy chains, and calponin [46]. While it's unclear whether pericytes utilize the same contraction mechanisms as SMCs, they are thought to modulate CBF through contraction. Our data also revealed an increase in the prevalence of stress fibers in APPswe iPLCs. Stress fibers are linked with contractile activity in myofibroblasts and cardiomyocytes [47]. SMCs obtained from AD individuals have been reported to express higher levels of contractile proteins [48]. Moreover, Yilmaz-Ozcan and coworkers have demonstrated that a decrease in α-SMA expression levels reduces pericyte contraction in mouse brain [49]. These findings further support the hypothesis that there is a correlation between pericyte contraction and α-SMA expression. Previous publications have also shown that Erk and p38 MAPK pathways regulate myosin light chain phosphatase, thereby contributing to sustained smooth muscle contraction [50, 51]. Our examination of Erk phosphorylation following exposure to vasoconstrictor ET-1 revealed enhanced Erk pathway activation in APPswe iPLCs. This suggests that the hypercontractility phenotype, characterized by a steeper slope and prolonged recovery time, may stem from amplified Erk signaling. However, additional evidence is required to firmly establish this conclusion.

The hypercontractile phenotype observed in APPswe iPLCs may also be linked to Aβ pathology. A previous study found that the accumulation of Aβ had a constricting effect on capillaries in patients with cognitive decline by eliciting the ET-1 signaling pathway in pericytes [52]. Similarly, in vitro human pericytes exhibited compromised contraction and relaxation after exposure to exogenous Aβ [26, 27]. Thus, the hypercontractile phenotype observed in APPswe iPLCs in our model is consistent with prior studies and could potentially cause decreased CBF in patients with AD. The hypercontractile phenotype correlates with elevated expression levels of contractile proteins, amplified Erk signaling, and Aβ secretion. Further research is required to fully understand the underlying mechanisms.

In dextran permeability studies, APPswe iPLCs exhibited a compromised ability to enhance the tightness of iEC layer. This effect could potentially result in BBB leakage, commonly seen in CAA patients, leading to neuroinflammation and accelerating the progression of AD [53]. However, the total levels of TJPs were higher in APPswe iPLCs. This suggests that the impaired ability of APPswe iPLCs to promote barrier formation cannot be explained by a degradation of the occludin and ZO-1 and may be attributed to differences in iPLC attachment or the involvement of other TJPs and/or signaling pathways. Moreover, APPswe iPLCs secreted significantly higher levels of the chemokine MCP-1. Overexpression of MCP-1 and VCAM-1 in the endothelium can cause monocytes to adhere strongly to the endothelial layer [54, 55]. This phenomenon has been linked with endothelial barrier dysfunction [56, 57]. Thus, our in vitro models containing APPswe iPLCs successfully replicate some features of CAA.

Angiogenesis plays a crucial role in the development and adulthood. Impaired angiogenesis can decrease vessel density, reduce CBF and nutrient transport, and thus potentially worsen the progression of AD. Previous studies indicated that reduced capillary density may result from both vessel degeneration and insufficient angiogenesis due to a lack of angiogenic factors. To validate the role of iPLCs in vessel formation, we compared iEC cultures alone to those exposed to an angiogenic cocktail and those co-cultured with iPLCs. In our hands, the co-culture with control iPLCs showed a similar enhancement of tube-like structure formation as the cocktail of angiogenic factors, suggesting that iPLCs were able to stimulate angiogenesis. The iPLC-conditioned medium alone did not have the same effect likely because the concentration of angiogenic factors was too low. Indeed, we could not detect the major angiogenic factor VEGF-A in the conditioned medium by ELISA. Nevertheless, iPLCs did produce VEGF-A protein as detected by ELISA in the whole-cell lysates, and we can speculate that locally released VEGF-A and possibly other angiogenic factors can reach sufficiently high levels to promote angiogenesis. We observed that co-cultured iPLCs had an even stronger impact on the number of master segments and meshes compared to the angiogenic cocktail. Our results do not exclude the stabilizing effect of pericytes on newly formed vessels.

Interestingly, APPswe iPLCs induced slightly less complex tube structures than control cells. We identified angiogenesis-related genes that were significantly reduced in APPswe iPLCs at the mRNA level (Fig. 3 D and 4 D), potentially linking them to the observed phenotype. These included *VEGFA* and its receptor *KDR*, *IL6R,* and *ITGA6.* IL-6/IL6R signaling has been reported to regulate endothelial cell proliferation and migration [58–60]. ITGA6 protects established endothelial tubes by regulating CXCR4 expression [61]. Our study reveals transcriptional and functional abnormalities in APPswe iPLCs hindering their ability to support angiogenesis and suggests potential molecular targets for future research.

In conclusion, our study shows that APPswe iPLCs induce CAA-like changes, leading to increased BBB permeability and defective angiogenesis and vasoconstriction in vitro. Utilizing human iPSC-derived models of brain vascular cells may enhance our understanding of various diseases, including CAA, and help identify critical pathways and develop novel therapies. Given the high prevalence of vascular dysfunction in AD, combination drugs targeting both Aβ pathology and vascular dysfunction could be a promising therapeutic strategy.

## Conclusions

Using the established iPLC protocol, our study reaffirms that these cells express classic in vivo pericyte markers. We conducted permeability, angiogenesis, and contraction assays to demonstrate that these cells also replicate in vivo pericyte functions. Further, we explored how iPLCs contribute to vascular dysfunction in AD by using lines from individuals with APPswe mutation. Our findings indicate that the APPswe mutation leads to functional defects and transcriptome changes in iPLCs. Notably, altered functions such as impaired barrier integrity, reduced angiogenesis support, and hypercontractility are linked to CAA pathology. This suggests a significant role for pericytes in CAA pathology, potentially exacerbating vessel destabilization and inducing vascular dysfunction in AD. Additionally, our RNA-seq results identified potential pathways that might contribute to these observed phenotypes. As a result, our study has provided an iPSC-derived, human-based platform for drug screening targeting pericytes and/or vascular endothelial cells in the future.

## Supplementary Information

Supplementary File 1. Figure 1. Characterization of pluripotency of Ctrl1, Ctrl2 and Ctrl7 iPSC cell lines (A) Representative immunocytochemistry images of OCT4, NANOG, TRA 1-81 and SSEA4 Ctrl1, Ctrl2 and Ctrl7. Scale bars, 100 µm. (B) Representative karyograms from Ctrl1, Ctrl2 and Ctrl7 showing normal euploid karyotypes (46,XX for Ctrl1, Ctrl2 and 46, XY for Ctrl7).

Supplementary File 2. Figure 2. Pluripotency and endothelial markers are not expressed on iPLCs. (A) The relative gene expression levels of pluripotency markers* NANOG*,* LIN28A *and* SOX2* were compared between iPLCs and iPSCs, quantified as fold changes relative to *GAPDH*. (B) The relative gene expression levels of ECs marker* CDH5* were compared between iECs, iPLCs and iPSCs, quantified as fold changes relative to *GAPDH*. (C) Relative gene expression levels of *PDGFRB* and *DLC1 *in iPLCs across Day 7, 21, 31, and 50. Expression levels are shown as fold change relative to *GAPDH*. The dots indicate the average values of technical replicates for each biological sample (lines, batches), with the color of the dots representing different lines. The data are presented as mean ± SD. Statistical analysis was performed using one-way ANOVA with Dunnett’s multiple comparison test. The significance levels are denoted as follows: *p < 0.05, **p < 0.01,***p < 0.001 and ****p < 0.0001

Supplementary File 3. Figure 3. DEGs of APPswe versus control iPLCs (A) Heat map depicting DEGs between control and APPswe iPLCs (cutoffs: Adjusted *p*‐value <0.05 and absolute log2 fold change >1.5). The analysis included seven control and three APPswe lines

Supplementary File 4.

Supplementary File 5.

## Data Availability

The raw sequencing data and metadata are accessible on the EU Open Research Repository (Pilot) in Zenodo, identified by 10.5281/zenodo.11488682 (https://doi.org/10.5281/zenodo.11488682). Please note that the raw RNA-seq data is kept strictly confidential to uphold patient privacy and confidentiality.

## Abbreviations

ATP: Adenosine triphosphate
α-SMA: Alpha-smooth muscle actin
AD: Alzheimer's disease
APPswe: Swedish mutation in the amyloid precursor protein
APOE: Apolipoprotein E
ADM: Astrocyte differentiation medium
Aβ: Beta-amyloid
BBB: Blood–brain barrier
CAA: Cerebral amyloid angiopathy
CBF: Cerebral blood flow
DEGs: Differentially expressed genes
Erk: Extracellular signal-regulated kinase (p44/42 MAPK)
ET: Endothelin
bFGF: Fibroblast growth factor 2
HRP: Horseradish peroxidase
iPSCs: Induced pluripotent stem cells
IPA: Ingenuity Pathway Analysis
ICAM: Intercellular adhesion molecule
IL: Interleukin
iECs: IPSC-endothelial cells
iPLCs: IPSC-pericyte-like cells
MCP-1/CCL2: Monocyte chemoattractant protein 1
NDM: Neural differentiation medium
NG2: Neural-glial antigen 2, proteoglycan
PMA: Phorbol 12-myristate 13-acetate
PDGFRβ: Platelet-derived growth factor receptor beta
PVDF: Polyvinylidene difluoride
PSEN1/2: Presenilin-1 and -2
RIPA: Radioimmunoprecipitation assay buffer
RANTES/CCL5: Regulated on activation, normal T cell expressed and secreted
RNA-seq: RNA sequencing
SMCs: Smooth muscle cells
S1P: Sphingosine-1-phosphate
TLR: Toll-like receptor
TJPs: Tight junction proteins
ETV2: Transcription factor E26 transformation-specific variant 2
TNFα: Tumor necrosis factor-alpha
VCAM: Vascular cell adhesion molecule
VEGF: Vascular endothelial growth factor
