# Supplementary figures and images for: Human iPSC-derived pericyte-like cells carrying APP Swedish mutation overproduce beta-amyloid and induce cerebral amyloid angiopathy-like changes
Source: Fluids Barriers CNS. 2024 Sep 27;21:78. doi: 10.1186/s12987-024-00576-y (PMC11438249; doi:10.1186/s12987-024-00576-y)

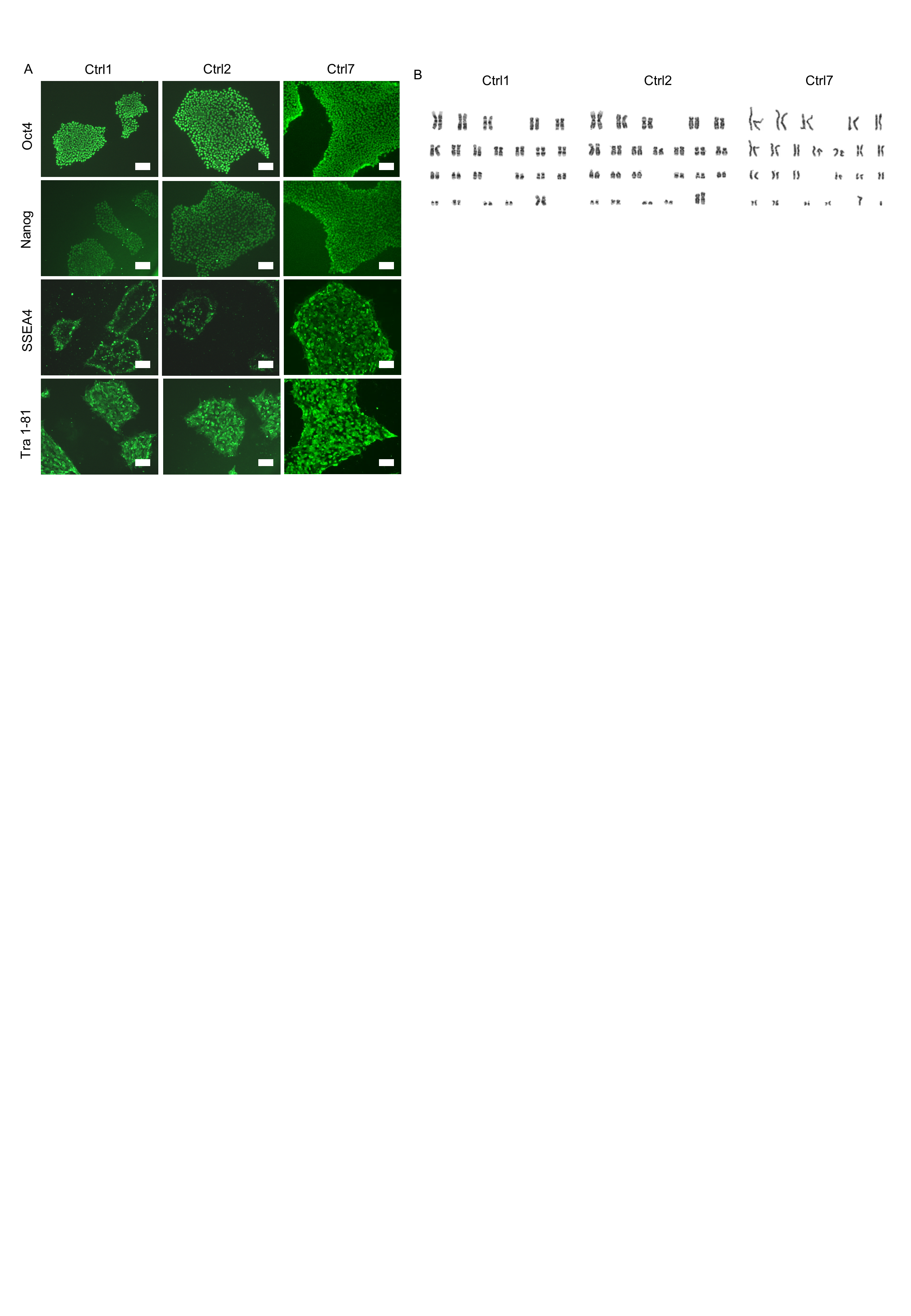

Supplement: Supplementary file 1 — Figure 1. Characterization of pluripotency of Ctrl1, Ctrl2 and Ctrl7 iPSC cell lines (A) Representative immunocytochemistry images of OCT4, NANOG, TRA 1-81 and SSEA4 Ctrl1, Ctrl2 and Ctrl7. Scale bars, 100 µm. (B) Representative karyograms from Ctrl1, Ctrl2 and Ctrl7 showing normal euploid karyotypes (46,XX for Ctrl1, Ctrl2 and 46, XY for Ctrl7). [file 12987_2024_576_MOESM1_ESM.tif]

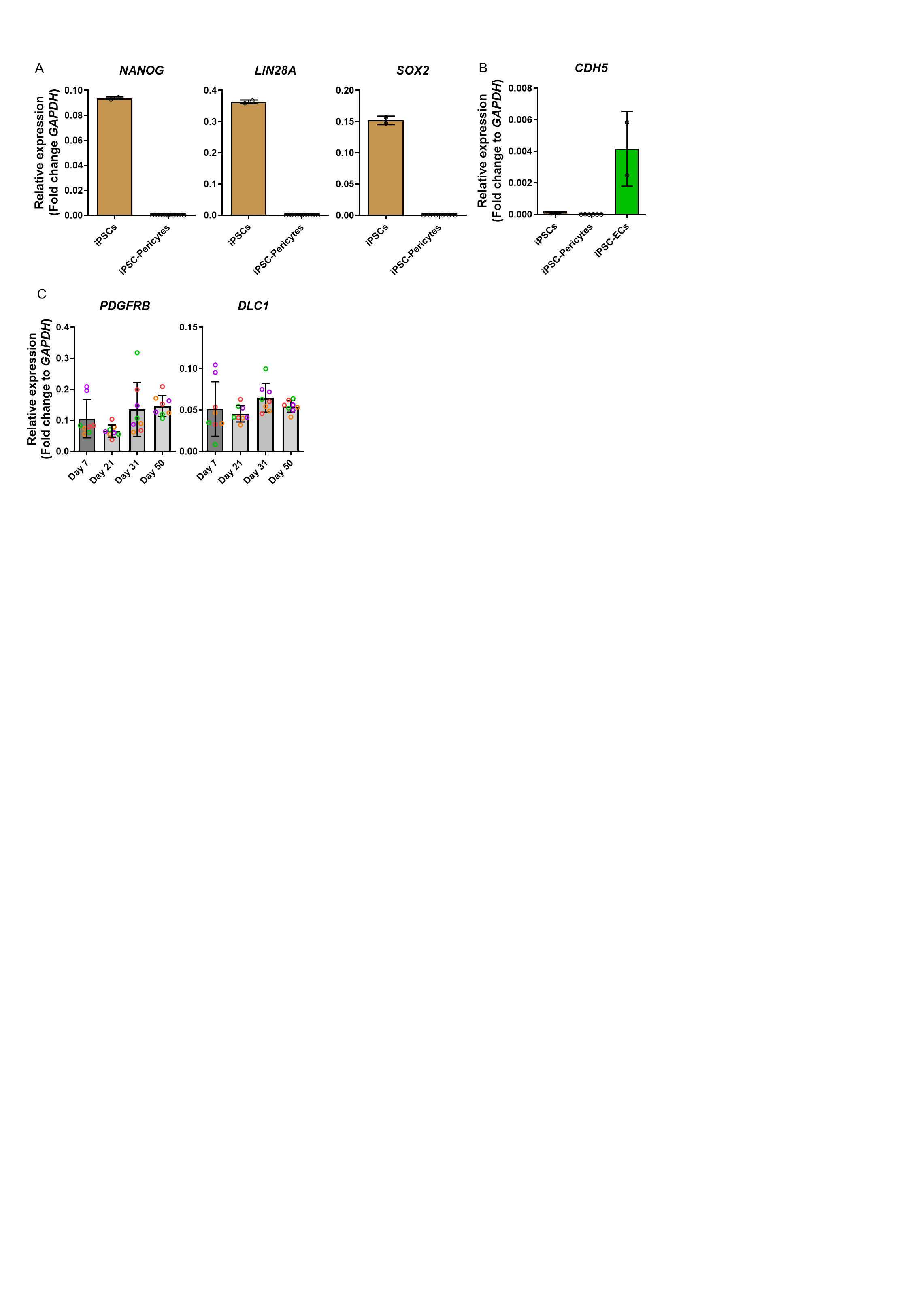

Supplement: Supplementary file 2 — Figure 2. Pluripotency and endothelial markers are not expressed on iPLCs. (A) The relative gene expression levels of pluripotency markers NANOG, LIN28A and SOX2 were compared between iPLCs and iPSCs, quantified as fold changes relative to GAPDH. (B) The relative gene expression levels of ECs marker CDH5 were compared between iECs, iPLCs and iPSCs, quantified as fold changes relative to GAPDH. (C) Relative gene expression levels of PDGFRB and DLC1 in iPLCs across Day 7, 21, 31, and 50. Expression levels are shown as fold change relative to GAPDH. The dots indicate the average values of technical replicates for each biological sample (lines, batches), with the color of the dots representing different lines. The data are presented as mean ± SD. Statistical analysis was performed using one-way ANOVA with Dunnett’s multiple comparison test. The significance levels are denoted as follows: *p < 0.05, **p < 0.01,***p < 0.001 and ****p < 0.0001 [file 12987_2024_576_MOESM2_ESM.tif]

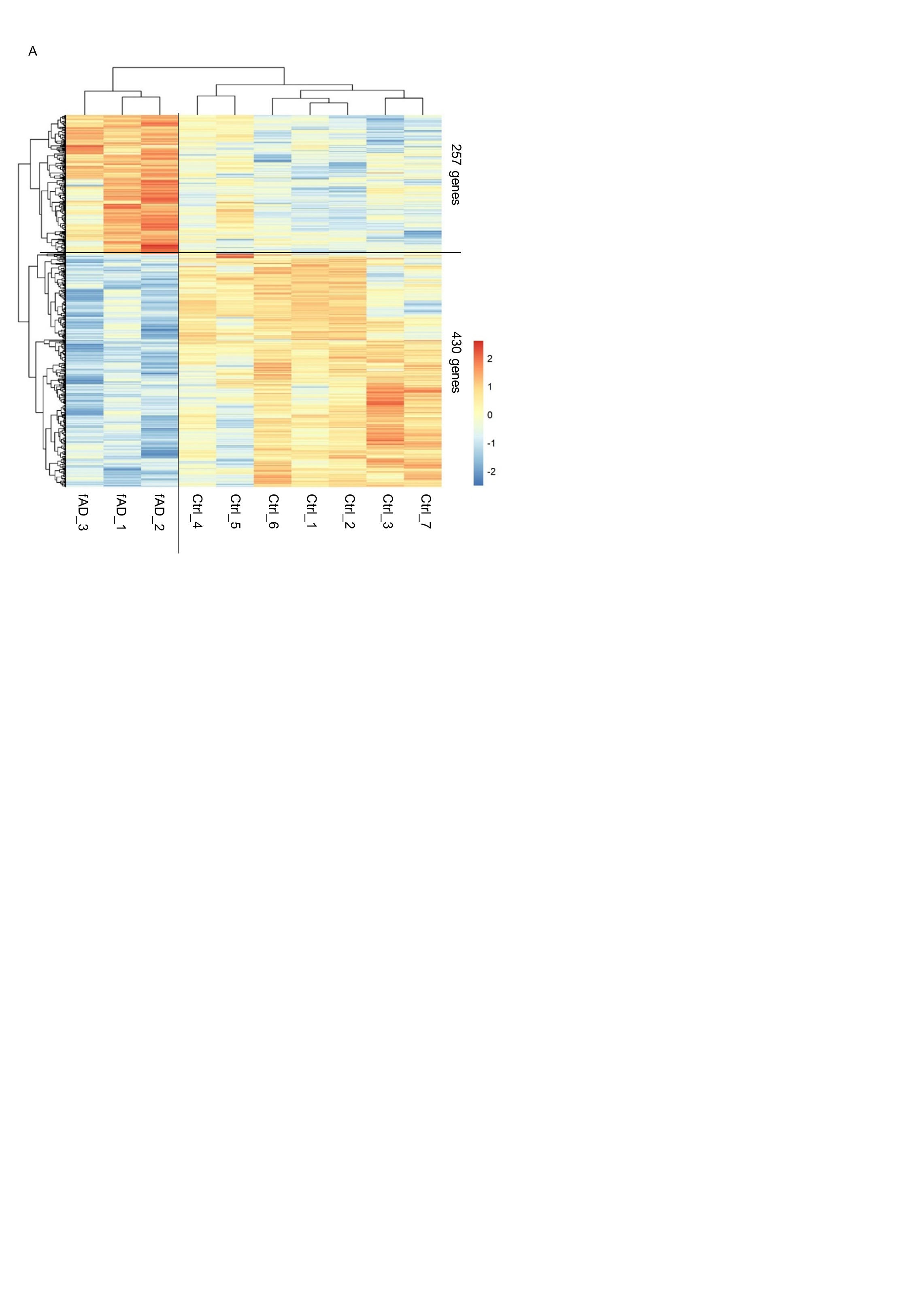

Supplement: Supplementary file 3 — Figure 3. DEGs of APPswe versus control iPLCs (A) Heat map depicting DEGs between control and APPswe iPLCs (cutoffs: Adjusted p‐value <0.05 and absolute log2 fold change >1.5). The analysis included seven control and three APPswe lines [file 12987_2024_576_MOESM3_ESM.tif]
