# Supplementary material for: Human iPSC-derived pericyte-like cells carrying APP Swedish mutation overproduce beta-amyloid and induce cerebral amyloid angiopathy-like changes
Source: Fluids Barriers CNS. 2024 Sep 27;21:78. doi: 10.1186/s12987-024-00576-y (PMC11438249; doi:10.1186/s12987-024-00576-y)

Blots for ZO-1 and Occludin expression quantification (Figure 1 J,K and 4 G) Batch#1&2

Merge with ladder images

Occludin Batch#1&2

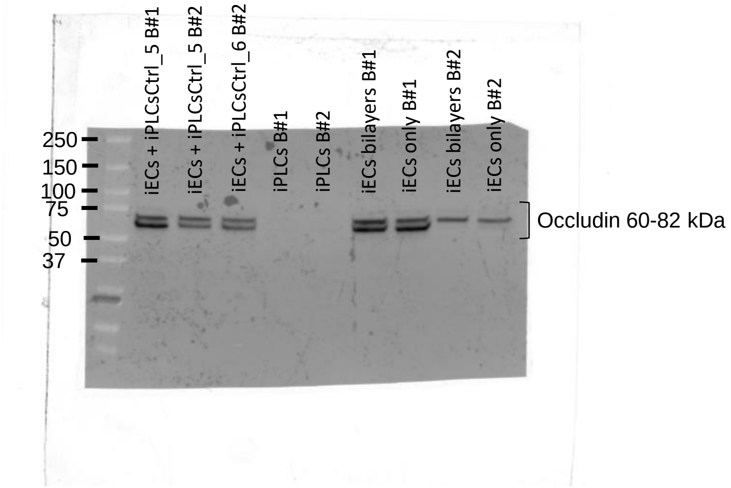

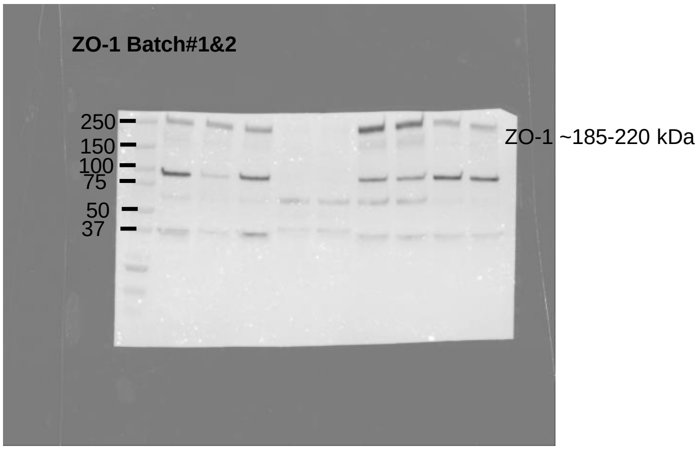

GAPDH Batch#1&2

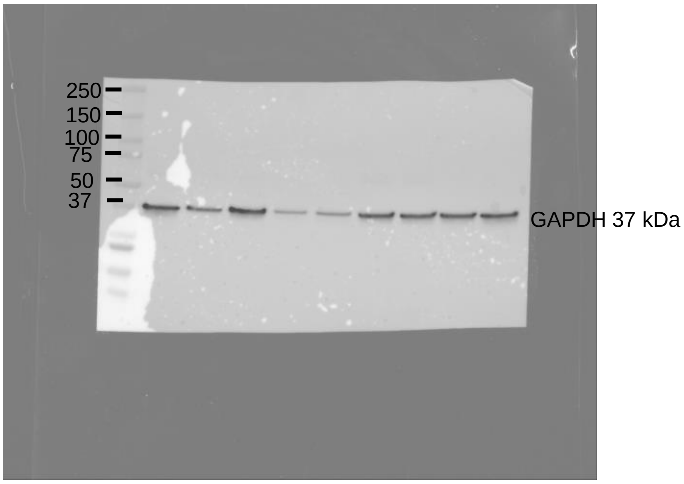

Images for quantification

Occludin Batch#1&2

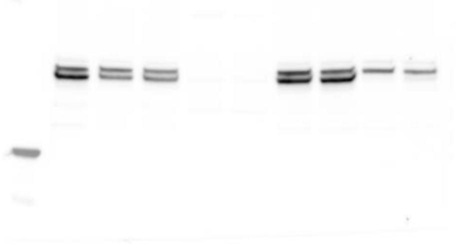

ZO-1 Batch#1&2

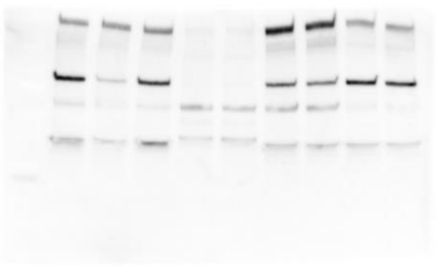

GAPDH Batch#1&2

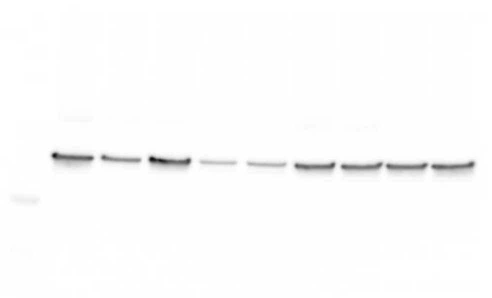

Blots for ZO-1 and Occludin expression quantification (Figure 1 J,K and 4 G) Batch#1

Merge with ladder images

Occludin Batch#1 iECs+iPLCs

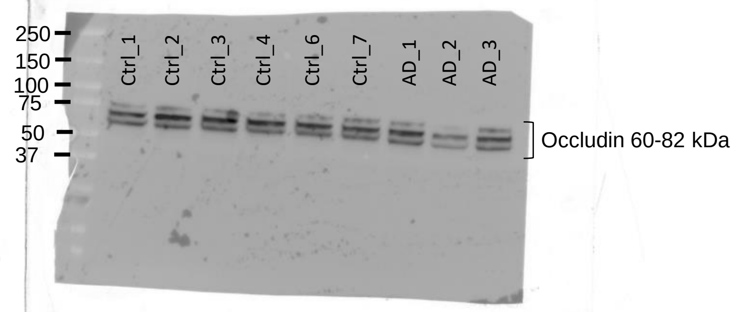

ZO-1 Batch#1

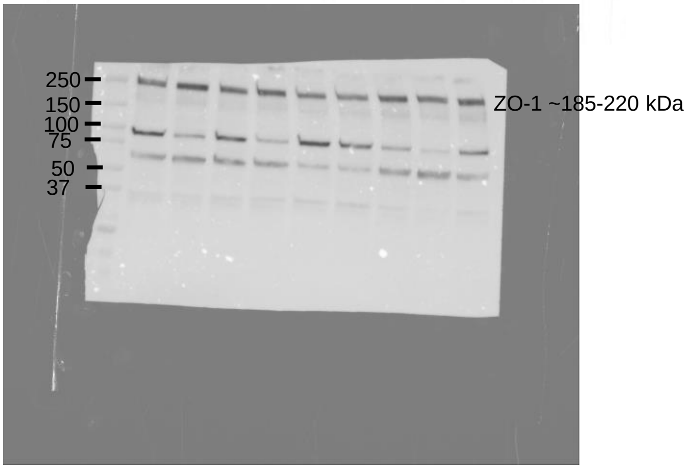

GAPDH Batch#1

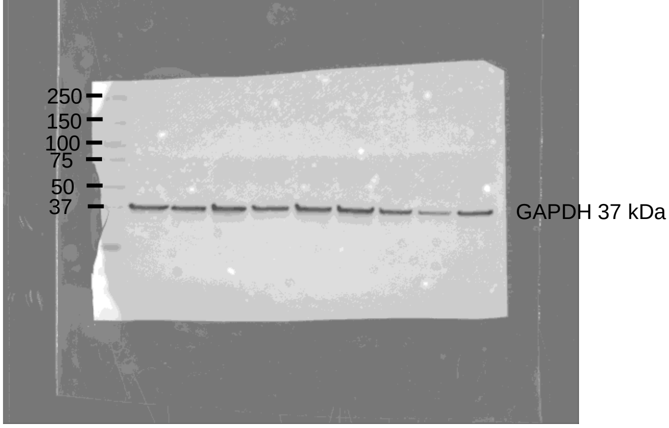

Images for quantification

Occludin Batch#1

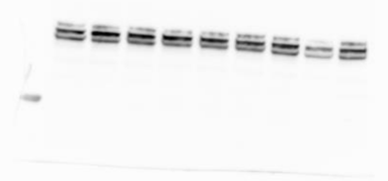

ZO-1 Batch#1

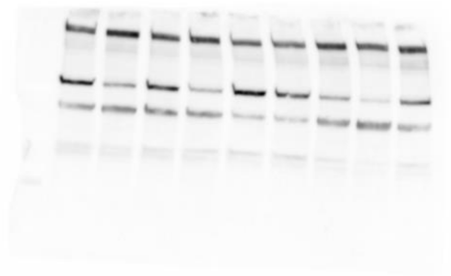

GAPDH Batch#1

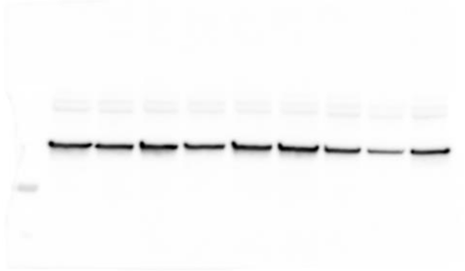

Blots for ZO-1 and Occludin expression quantification (Figure 1 J, K and 4 G) Batch#2

Merge with ladder images

Images for quantification

Occludin Batch#2 iECs+iPLCs

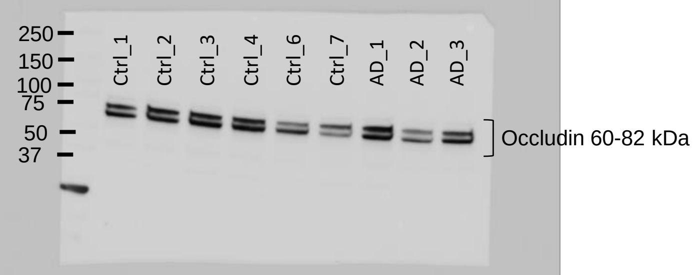

Occludin Batch#2

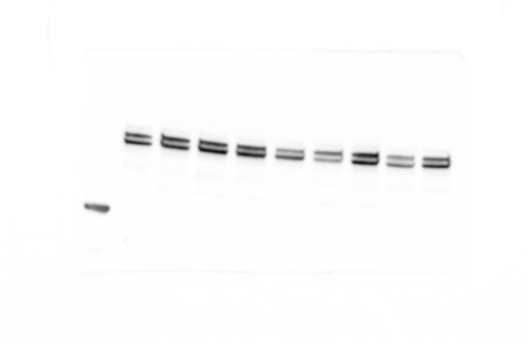

ZO-1 Batch#2

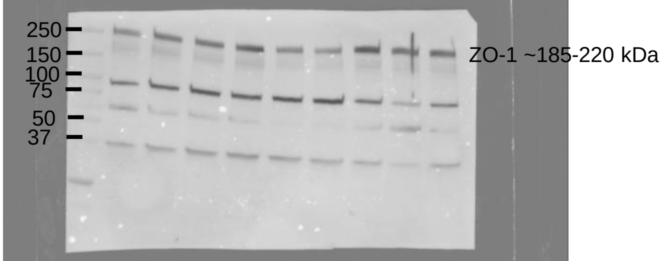

ZO-1 Batch#2

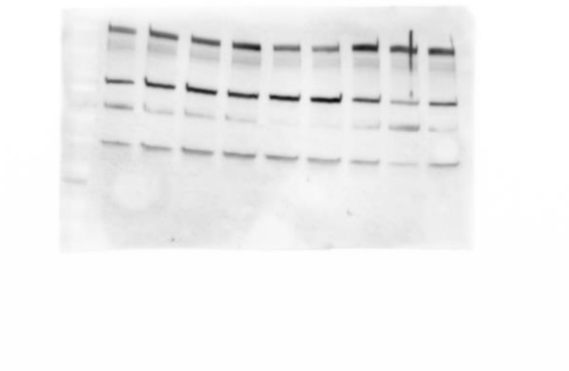

GAPDH Batch#2

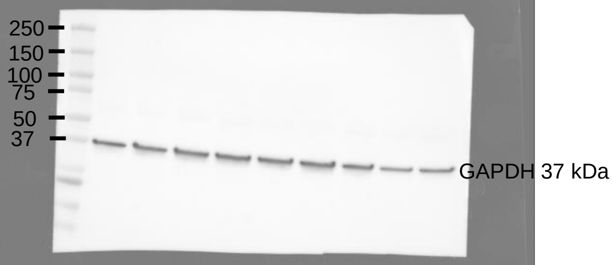

GAPDH Batch#2

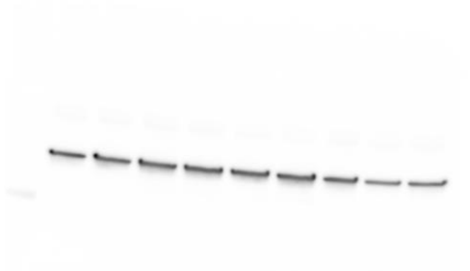

Blots for α-SMA expression quantification (Figure 2 C)

Merge with ladder images

Images for quantification

Batch 1

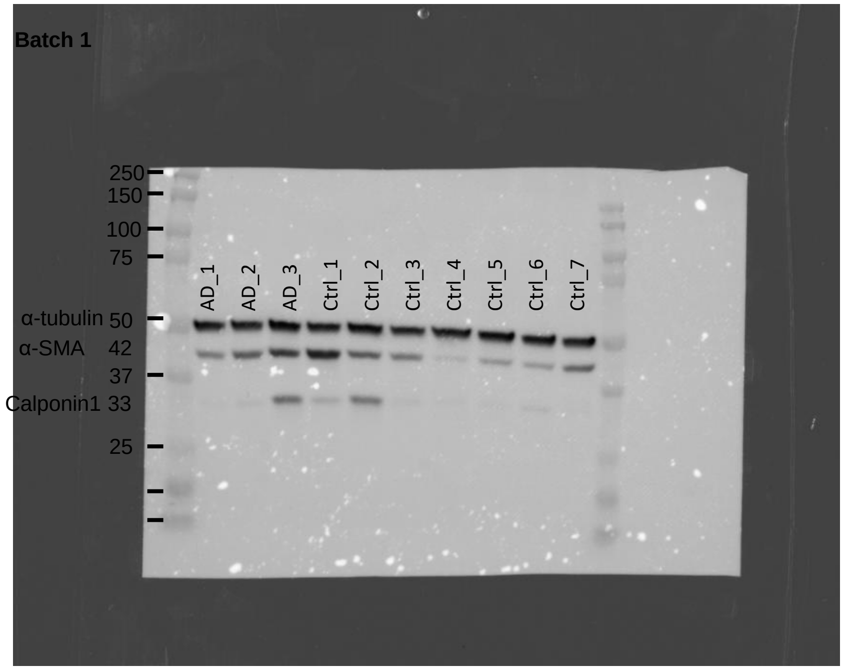

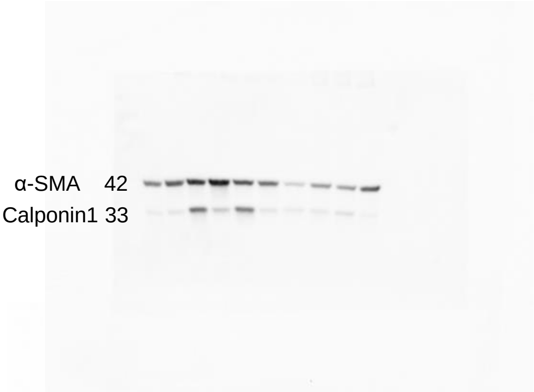

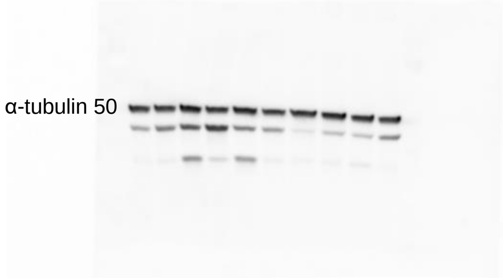

Batch 2

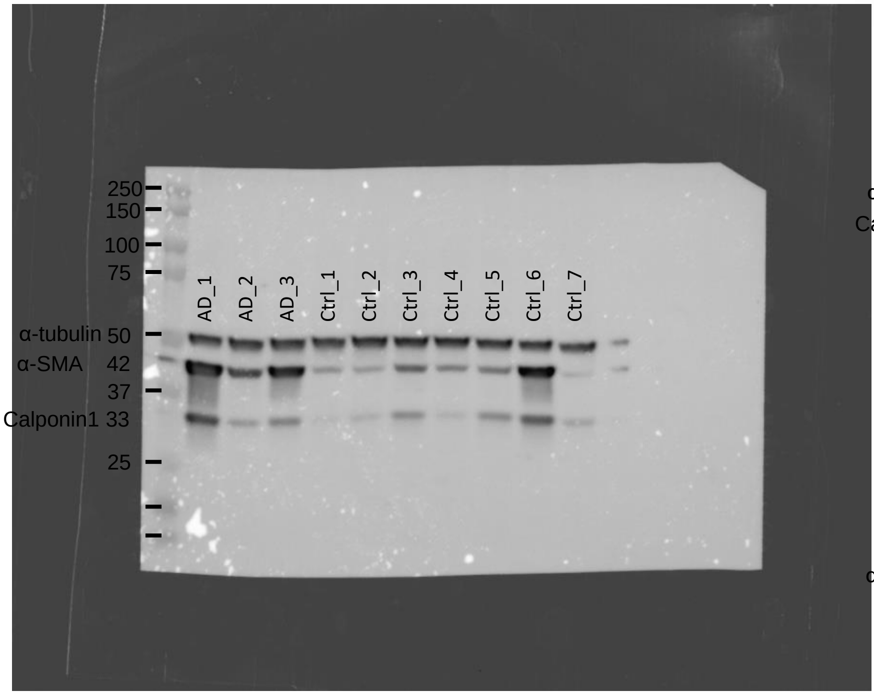

Batch 2

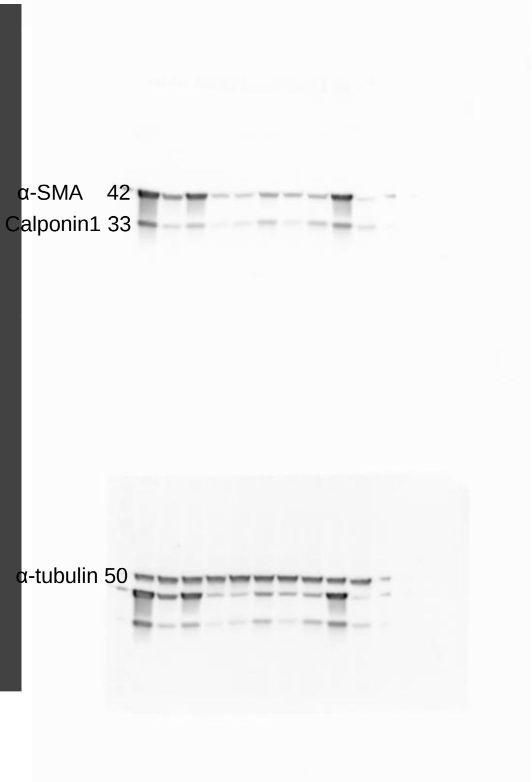

Blot for APP expression quantification (Figure 2 G)

Merge with ladder images

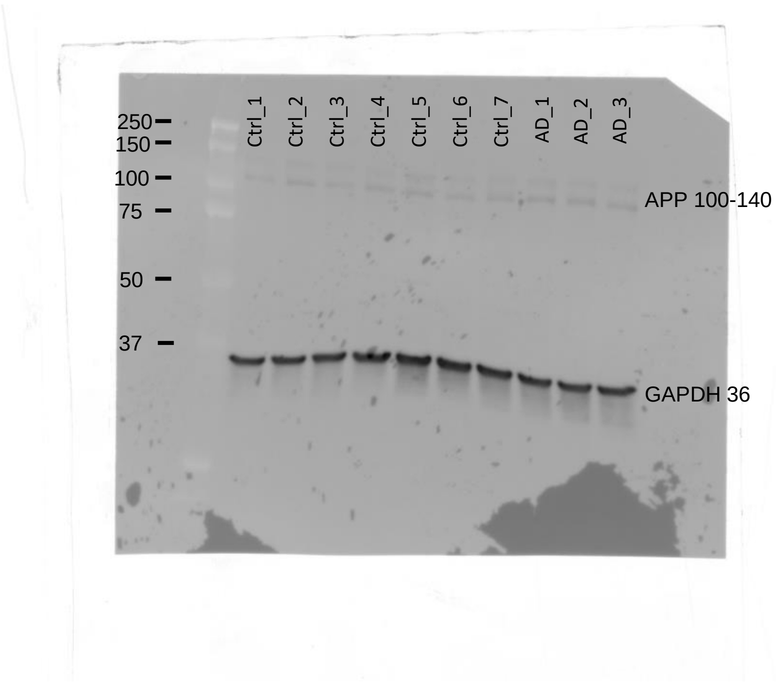

Images for quantification

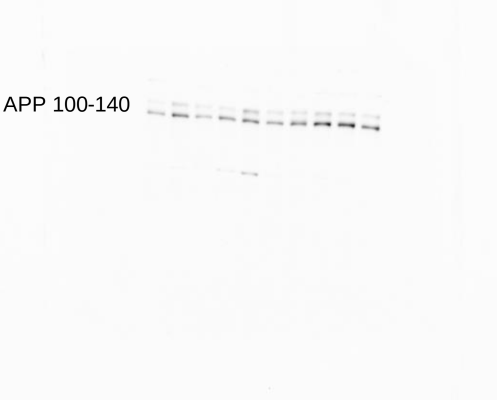

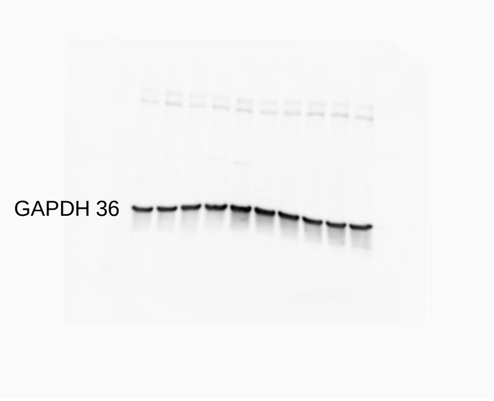

Blots for p-Erk, t-Erk expression quantification time points (Figure 5 F)

Merge with ladder images

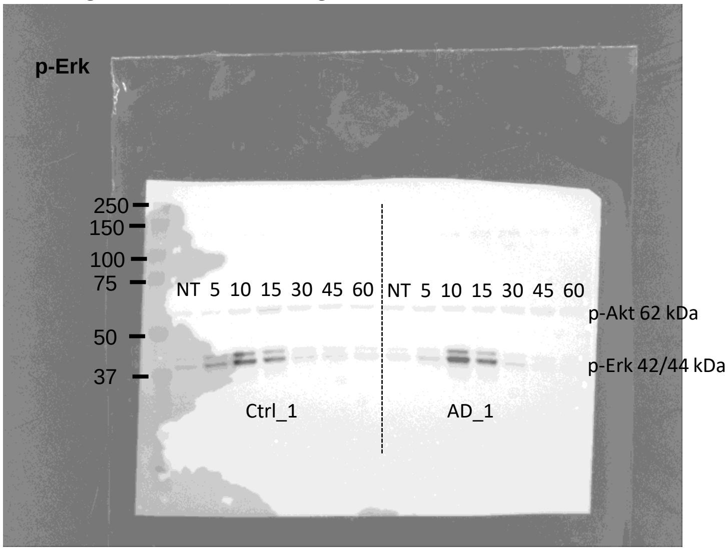

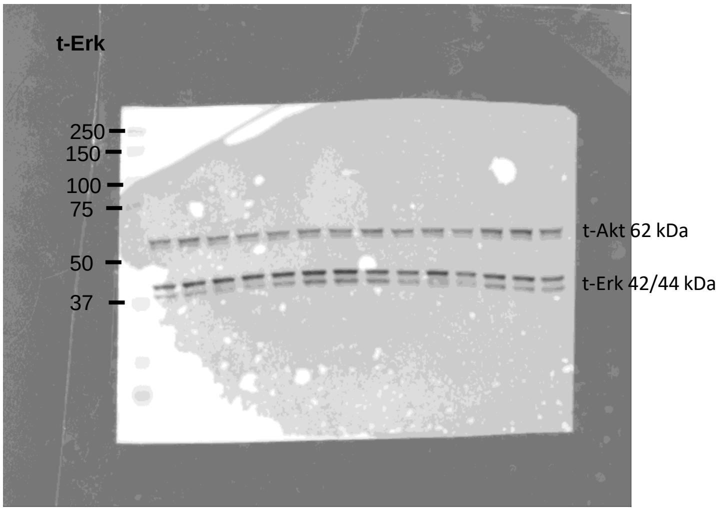

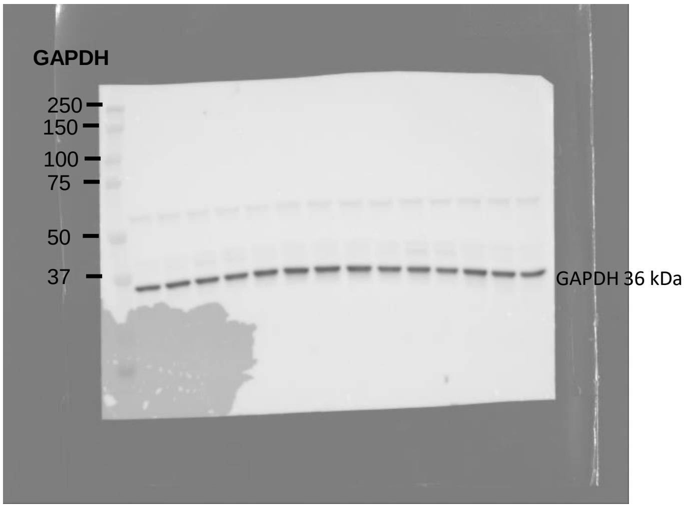

Images for quantification

p-Erk

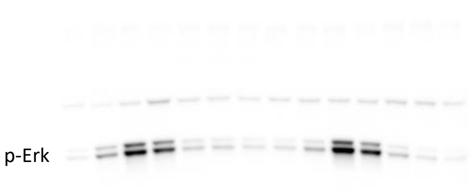

t-Erk

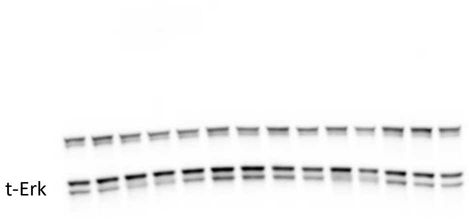

GAPDH

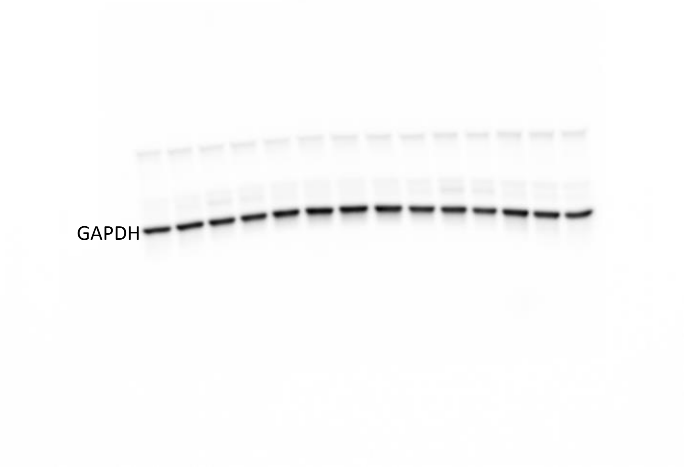

Blots for p-Erk, t-Erk expression quantification genotype (Figure 5 H,I) Batch#1 Membrane#1

Merge with ladder images

Images for quantification

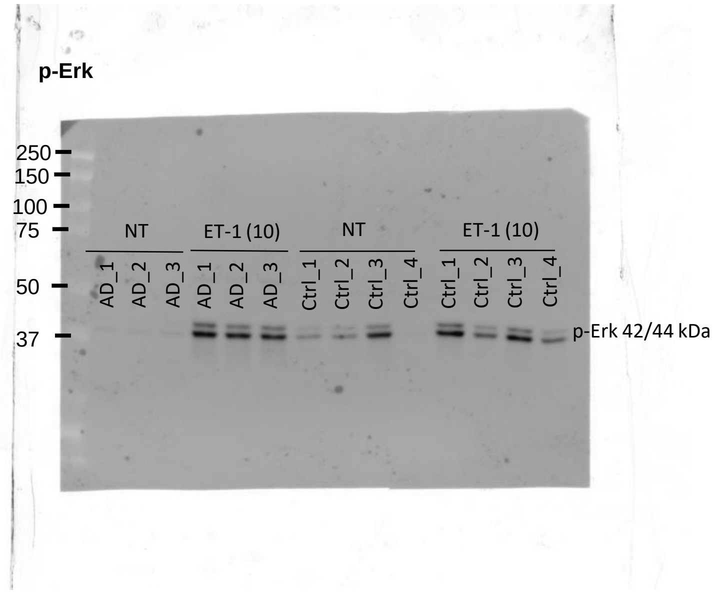

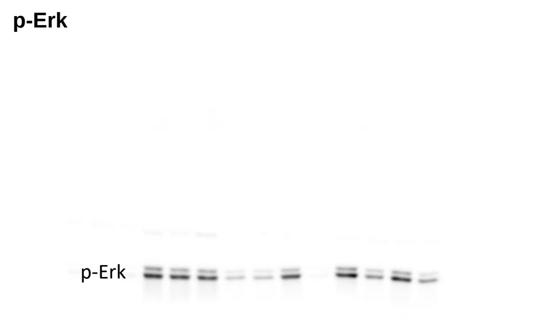

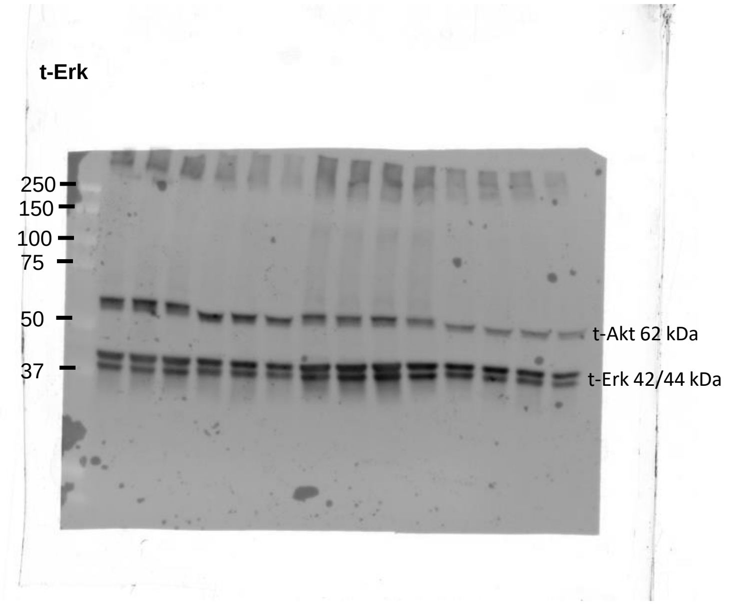

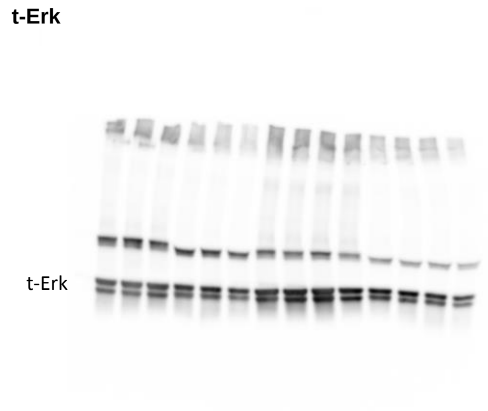

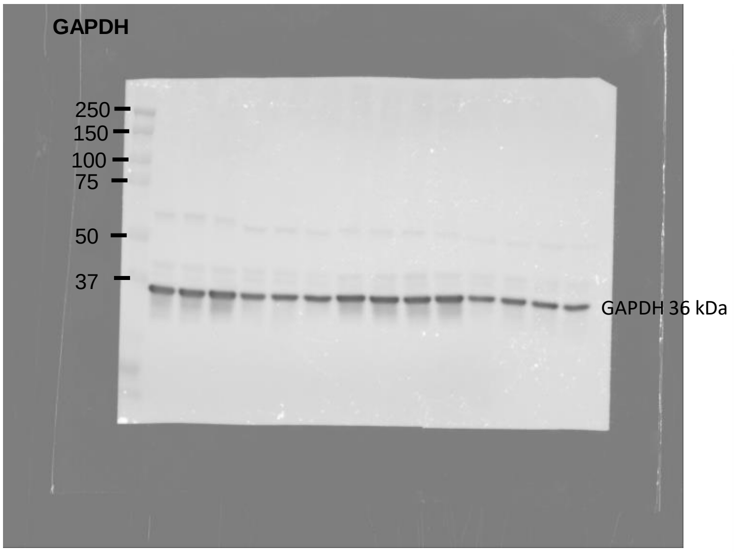

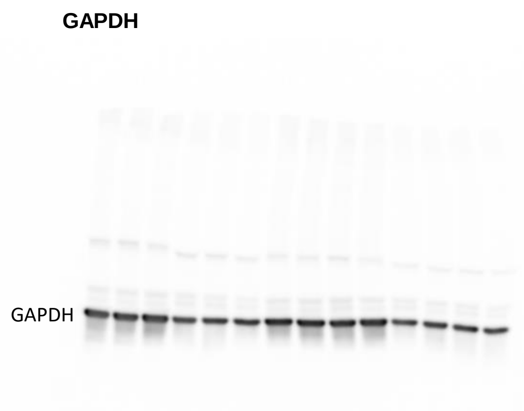

Blots for p-Erk, t-Erk expression quantification genotype (Figure 5 H,I) Batch#1 Membrane#2

Merge with ladder images

Images for quantification

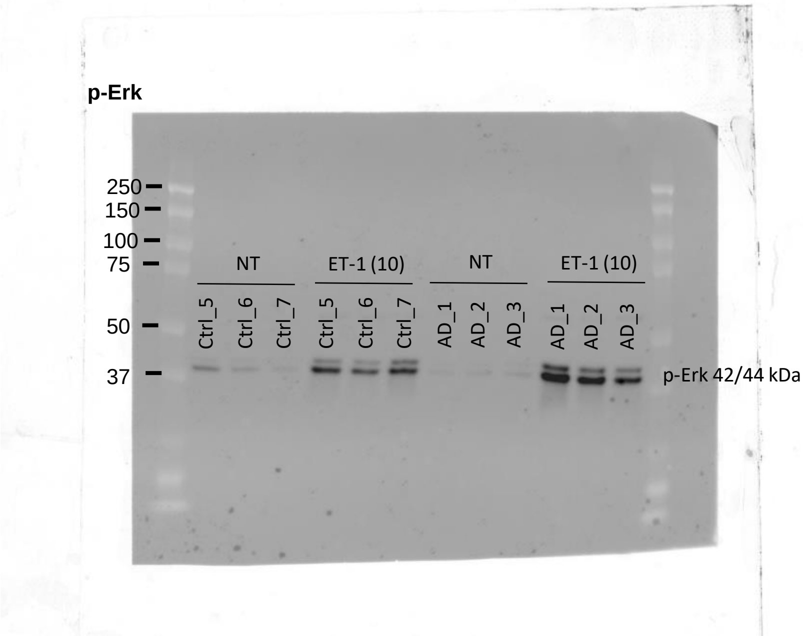

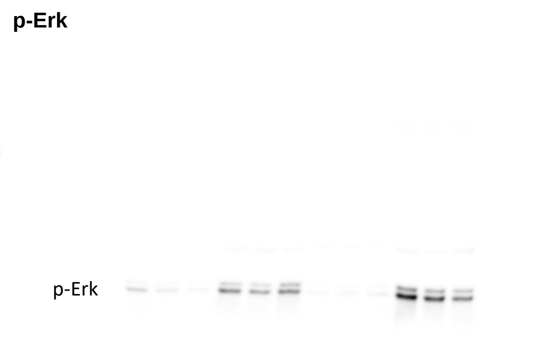

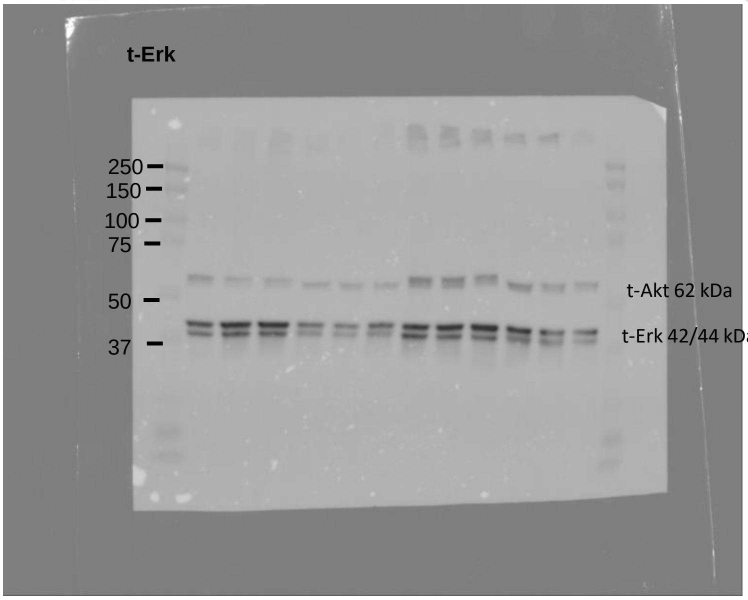

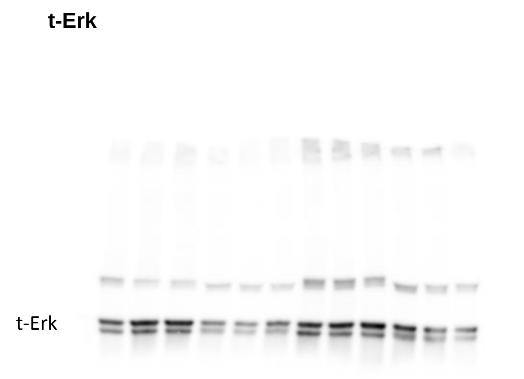

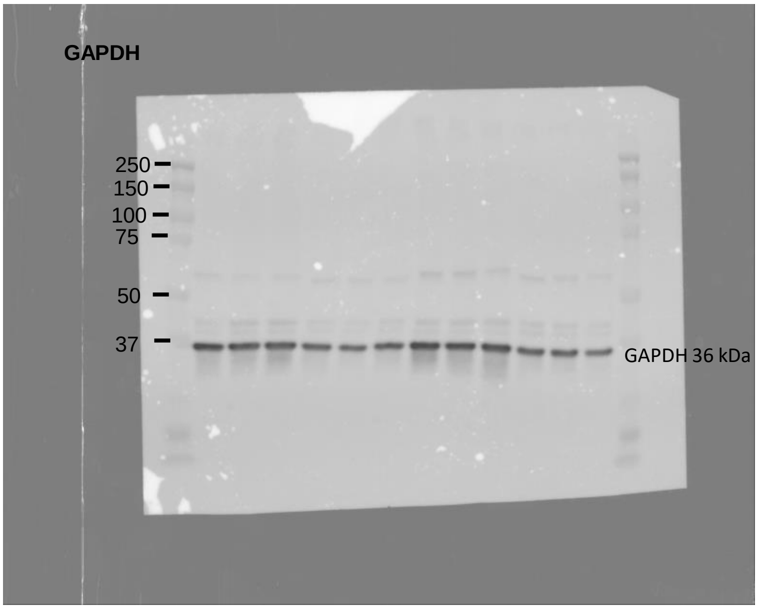

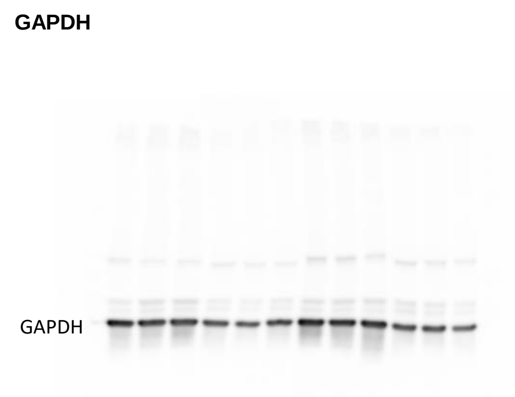

Blots for p-Erk, t-Erk expression quantification genotype (Figure 5 H,I) Batch#2 Membrane#2

Merge with ladder images

Images for quantification

p-Erk

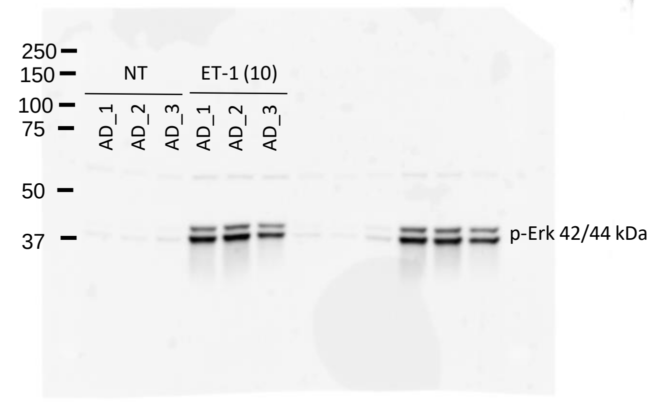

p-Erk

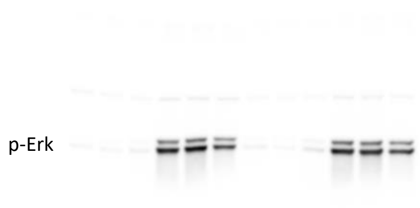

t-Erk

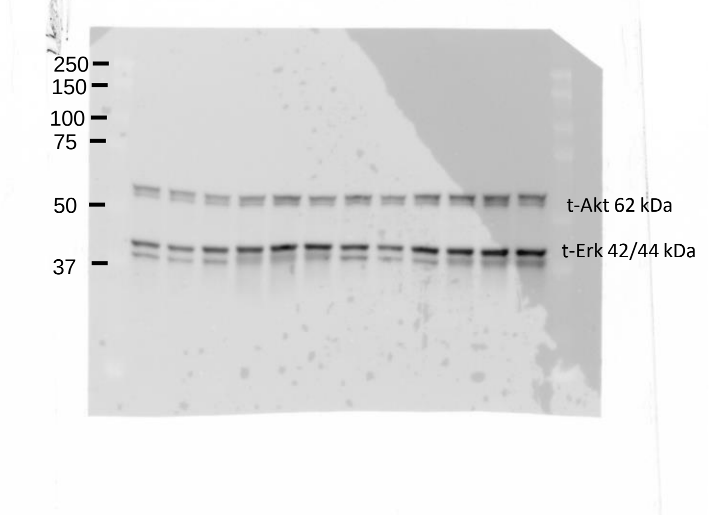

t-Erk

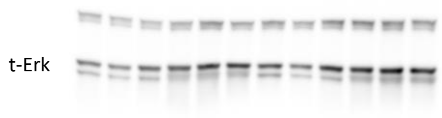

GAPDH

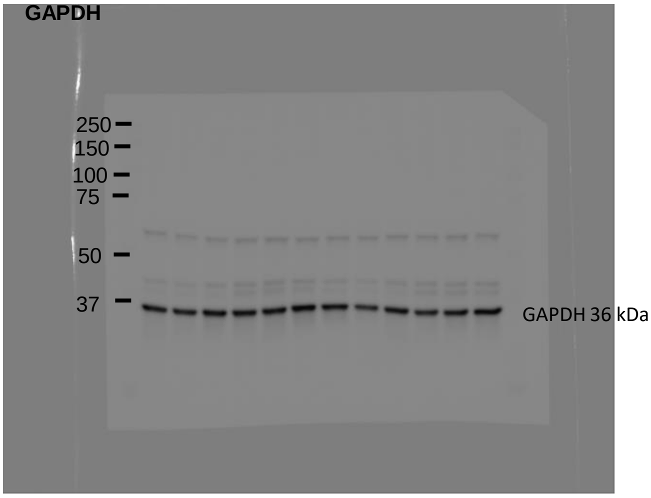

GAPDH

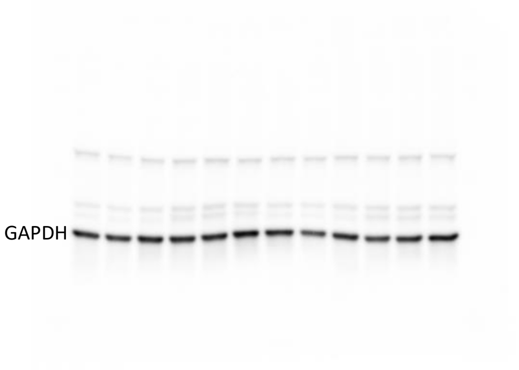

Supplement: Supplementary file 4 [file 12987_2024_576_MOESM4_ESM.pdf]
